# Brown banded bamboo shark (*Chiloscyllium punctatum*) shows high genetic diversity and differentiation in Malaysian waters

**DOI:** 10.1038/s41598-021-94257-7

**Published:** 2021-07-21

**Authors:** Kean Chong Lim, Amy Yee-Hui Then, Alison Kim Shan Wee, Ahemad Sade, Richard Rumpet, Kar-Hoe Loh

**Affiliations:** 1 Institute of Ocean and Earth Sciences, Universiti Malaya , 50603 Kuala Lumpur, Malaysia; 2 Institute of Biological Sciences, Universiti Malaya , 50603 Kuala Lumpur, Malaysia; 3 Guangxi Key Laboratory of Forest Ecology and Conservation, College of Forestry, Guangxi University, Nanning, 530004 China; 4 School of Environmental and Geographical Sciences, University of Nottingham Malaysia, 43500 Semenyih, Malaysia; 5 Department of Fisheries Sabah, 88624 Kota Kinabalu, Sabah Malaysia; 6 Fisheries Research Institute Sarawak, Department of Fisheries Malaysia, 93744 Kuching, Sarawak Malaysia

**Keywords:** Population genetics, Haplotypes

## Abstract

The demersal brown banded bamboo shark *Chiloscyllium punctatum* is a major component of sharks landed in Malaysia. However, little is known about their population structure and the effect of high fishing pressure on these weak swimming sharks. Both mitochondrial DNA control region (1072 bp) and NADH dehydrogenase subunit 2 (1044 bp) were used to elucidate the genetic structure and connectivity of *C. punctatum* among five major areas within the Sundaland region. Our findings revealed (i) strong genetic structure with little present day mixing between the major areas, (ii) high intra-population genetic diversity with unique haplotypes, (iii) significant correlation between genetic differentiation and geographical distance coupled with detectable presence of fine scale geographical barriers (i.e. the South China Sea), (iv) historical directional gene flow from the east coast of Peninsular Malaysia towards the west coast and Borneo, and (v) no detectable genetic differentiation along the coastline of east Peninsular Malaysia. Genetic patterns inferred from the mitochondrial DNA loci were consistent with the strong coastal shelf association in this species, the presence of contemporary barriers shaped by benthic features, and limited current-driven egg dispersal. Fine scale population structure of *C. punctatum* highlights the need to improve genetic understanding for fishery management and conservation of other small-sized sharks.

## Introduction

Sharks are generally highly vulnerable to overexploitation due to their life history strategies, such as late maturity and low fecundity^1–5^. Overexploitation has been shown to reduce genetic diversity and increase extinction risk especially for small populations^6^; therefore understanding the genetic structure and migration patterns or gene flow of shark species are essential to inform effective management and conservation plans. Earlier shark genetic studies had been largely focused on marine neritic shark species, that are charismatic, economically important and of conservation interest^5^, e.g. blacktip shark *C. limbatus*^7^, hammerhead shark *Sphyrna lewini*^8^, whale shark *Rhincodon typus*^9^ and great white shark *Carcharodon carcharias*^10–15^. Studies on small-sized benthic coastal sharks have increased in recent years in part due to their catch prominence and importance in coastal fisheries, both globally and in Southeast Asia, e.g. whitespotted bambooshark *Chiloscyllium plagiosum*^16^, nurse shark *Ginglymostoma cirratum*^17^, whitetip reef shark *Triaenodon obesus*^18^, leopard shark *Triakis semifasciata*^19^ and common smoothhound *Mustelus mustelus*^20^.

One such group of sharks is the longtail carpet shark or bamboo shark from the family Hemiscyllidae (order Orectolobiformes). The family Hemiscyllidae comprises two genera: *Hemiscyllium* Müller & Henle, 1838 (nine species) which is confined to the Australia-New Guinea region and Indonesia, and the Indo-Pacific genus of *Chiloscyllium* Müller & Henle, 1837 (eight species)^21^. These bottom-living sharks are known to be weak swimmers^22^ with great camouflage ability, allowing them to adapt to demersal habitat by hiding around crevices^23^. They live either solitarily or within a group and appear to be strongly territorial. They usually deposit egg cases on the sea floor (sheltered or deserted), with some cases attached to benthic marine plants^23^. Currently, their fecundity in the wild is still unknown, but studies on captive individuals suggested that they produce annually less than 30 egg cases that require a three-month incubation period^24–27^. To the best of our knowledge, no movement study on this animal exists. Dispersal is likely very restricted due to their oviparous nature^28^, thus rendering them highly vulnerable to localized exploitation and habitat destruction.

Presently, four species of *Chiloscyllium* are found in Malaysia: Indonesian bamboo shark *C. hasseltii* Bleeker, 1852; slender bamboo shark *C. indicum* (Gmelin, 1789); whitespotted bamboo shark *C. plagiosum* (Bennett, 1830); and brown banded bamboo shark *C. punctatum* Müller & Henle, 1838. A previous record of grey bamboo shark *C. griseum* Müller & Henle, 1838 in Malaysian waters was a misidentification of *C. hasseltii*^Ahmad, personal communication^. The brown banded bamboo shark *C. punctatum* has been classified as Near Threatened under the IUCN Red List but may be uplisted to Vulnerable in the near future in Southeast Asia due to high fishing pressure^29^. It is the top ten shark species landed by numbers in commercial fisheries across Malaysia^30^. This species makes up almost half of all shark individuals caught using bottom trawl fishing vessels in the west coast of Peninsular Malaysia^31^. Approximately 40% of *C. punctatum* landed in commercial fishing gears comprised of immature individuals, with higher percentage in the west than the east coasts of Peninsular Malaysia^31,32^^,Lim, unpublished data^. Among similar-sized sharks, *C. punctatum* has relatively lower commercial value, fetching an ex-vessel price of USD 0.20–0.60 per kg. Many artisanal fishers in Malaysia generally do not discard any of their non-trash fish catches; when they do, bamboo sharks are among those selectively discarded^Then, unpublished data^. No formal fishery assessment exists for this species (or other sharks) in Malaysia, and the effect of present fishing level on the animal is unknown.

One of the priorities for *C. punctatum* includes clarifying the population substructure due to concerns of population fragmentation^29^ likely due to being a weak swimmer and limited potential for dispersal. Genetic tools have been used to identify population vulnerability or risk of local extinction based on their genetic diversity and structure as well as connectivity among populations^33–35^. Intra-specific genetic studies on small-sized coastal sharks and rays with limited vagility showed that they display high genetic population differentiation across ocean basins due to environmental barriers (nurse shark *Ginglymostoma cirratum*^17^), vicariance and dispersal events (maskray *Neotrygon kuhlii*^36^), or a combination of both (small-spotted catshark *Scyliorhinus canicula*^37^). On the other hand, studies of these small coastal species along continuous continental coastlines either displayed clear genetic differentiation (leopard shark *Triakis semifasciata*^19^, short-tailed stingray *Dasyatis brevicaudata*^38^) or lack of genetic structure (milk shark *Rhizoprionodon acutus*^39^); reasons for these pattern variations included differences in habitat preferences, site fidelity, geographical distance, and physical barriers to movement. Despite growing concerns of shark exploitation in Malaysia as one of the top shark-fishing nation globally^40^, genetic studies of local shark populations, including *C. punctatum* are almost non-existent (see Dudgeon et al.^41^ for exception).

The genetic structure of any organism is influenced not only by the ecological processes but also the geological history^42–46^. Sundaland, a shelf within the biodiverse Indo-West Pacific region that extends from Thailand southwards primarily covering Malaysia and Indonesia, has undergone dramatic plate tectonic evolution forming episodic submergence and exposure of the shelf^47,48^. These series of events had affected distributions of local organisms (ranging from terrestrial to marine environment) in multiple ways due to different population dispersal patterns (e.g. Lim et al.^45^; Tan et al.^46^; Reid et al.^49^; Polgar et al.^50^; Leonard et al.^51^; Ma et al.^52^; Mason et al.^53^; Crandall et al.^54^). In relation to elasmobranch populations, the Sunda Shelf appeared to be acting as biogeographic barrier for wedgefish *Rhynchobatus australiae*^55^ but not for spottail shark *Carcharhinus sorrah*^56^. However finer-scale seascape features that may affect genetic structuring of sharks and rays within the Sundaland region had not been investigated previously.

The aim of the present study is therefore to assess the degree of genetic diversity and differentiation of *C. punctatum* in Malaysia, specifically along coastal Peninsular Malaysia and northern Malaysian Borneo coastline. Based on the biogeographic history of the Sundaland region, we hypothesized that there are at least two distinct local *C. punctatum* populations, with little genetic mixing between populations from either side of the peninsular (northern end of the Sunda Shelf barrier) and across the Karimata Strait that separates the peninsular from Borneo. Furthermore, we tested to see if observed population genetic differentiation can be explained by simple geographical distance or otherwise. In addition, we also assessed the gene flow directionality between distinctive populations to infer putative dispersal pattern. Knowledge of fine scale genetic structuring of *C. punctatum* provides the spatially relevant information for appropriate national fisheries management and conservation of this highly fished shark and likely of other sharks sharing similar life history strategies.

## Results

A total of 2116 base pair sequences hereby named as ND2CR (concatenated between 1072 bp for CR and 1044 bp for ND2were successfully amplified for all 135 *C. punctatum* samples. A total of 70 unique haplotypes and 63 polymorphic sites were detected, in which 40 sites were found in the CR region and the rest were found in the ND2 region (Supplementary Tables S1 and S2). Most of the identified haplotypes (70%) was found only in a single individual, while the rest was represented by two to 17 individuals. In terms of genetic diversity, ND2 gene showed lower range (haplotype diversity: 0.58–0.71, nucleotide diversity: 0.0009–0.0026) compared to CR (haplotype diversity: 0.63–0.90, nucleotide diversity: 0.0021–0.0076) (Table 1). When the two markers were concatenated, the range of haplotype diversity increased (0.76–0.98) (Table 1).

**Table 1:** Genetic diversity of *Chiloscyllium punctatum* according to area and markers. *N* = number of samples, *k* = number of polymorphic sites, *N*_*ha*_ = number of haplotypes, *ha*_=_ haplotype diversity, π = nucleotide diversity, D = Tajima’s D test, F_S_ = Fu’s F_S_ test.

Area	*N*	*k*	*N*_*ha*_	*ha*	π	D	Fs
**CR**							
West Peninsular	30	13	13	0.89 ± 0.03	0.0028 ± 0.0017	− 0.35	− 4.38*
East Peninsular	25	11	13	0.90 ± 0.04	0.0021 ± 0.0014	− 0.73	− 7.02*
Sarawak	26	18	15	0.90 ± 0.05	0.0046 ± 0.0026	0.15	− 4.34*
Western Sabah	25	19	7	0.63 ± 0.10	0.0053 ± 0.0029	0.43	2.87
Eastern Sabah	29	20	10	0.87 ± 0.03	0.0076 ± 0.0040	2.02	2.28
Total	135	40	52	0.95 ± 0.01	0.0086 ± 0.0004		
**ND2**							
West Peninsular	30	6	5	0.71 ± 0.05	0.0023 ± 0.0014	1.71	1.98
East Peninsular	25	5	6	0.68 ± 0.08	0.0009 ± 0.0007	− 0.92	− 2.23*
Sarawak	26	6	6	0.63 ± 0.09	0.0018 ± 0.0012	0.58	− 0.00
Western Sabah	25	10	7	0.70 ± 0.08	0.0026 ± 0.0016	0.03	0.13
Eastern Sabah	29	6	3	0.58 ± 0.05	0.0026 ± 0.0016	2.24	5.32
Total	135	23	19	0.85 ± 0.02	0.0041 ± 0.0002		
**ND2CR**							
West Peninsular	30	19	18	0.94 ± 0.02	0.0026 ± 0.0014	0.41	− 6.17*
East Peninsular	25	16	20	0.98 ± 0.02	0.0015 ± 0.0009	− 0.88	− 17.94*
Sarawak	26	24	18	0.95 ± 0.03	0.0033 ± 0.0018	0.29	− 5.85*
Western Sabah	25	29	10	0.76 ± 0.08	0.0040 ± 0.0021	0.30	1.79
Eastern Sabah	29	26	11	0.89 ± 0.03	0.0051 ± 0.0027	2.24	2.86
Total	135	63	70	0.97 ± 0.01	0.0063 ± 0.0002		

*Represent significant difference at p < 0.05.

**Figure 1:**
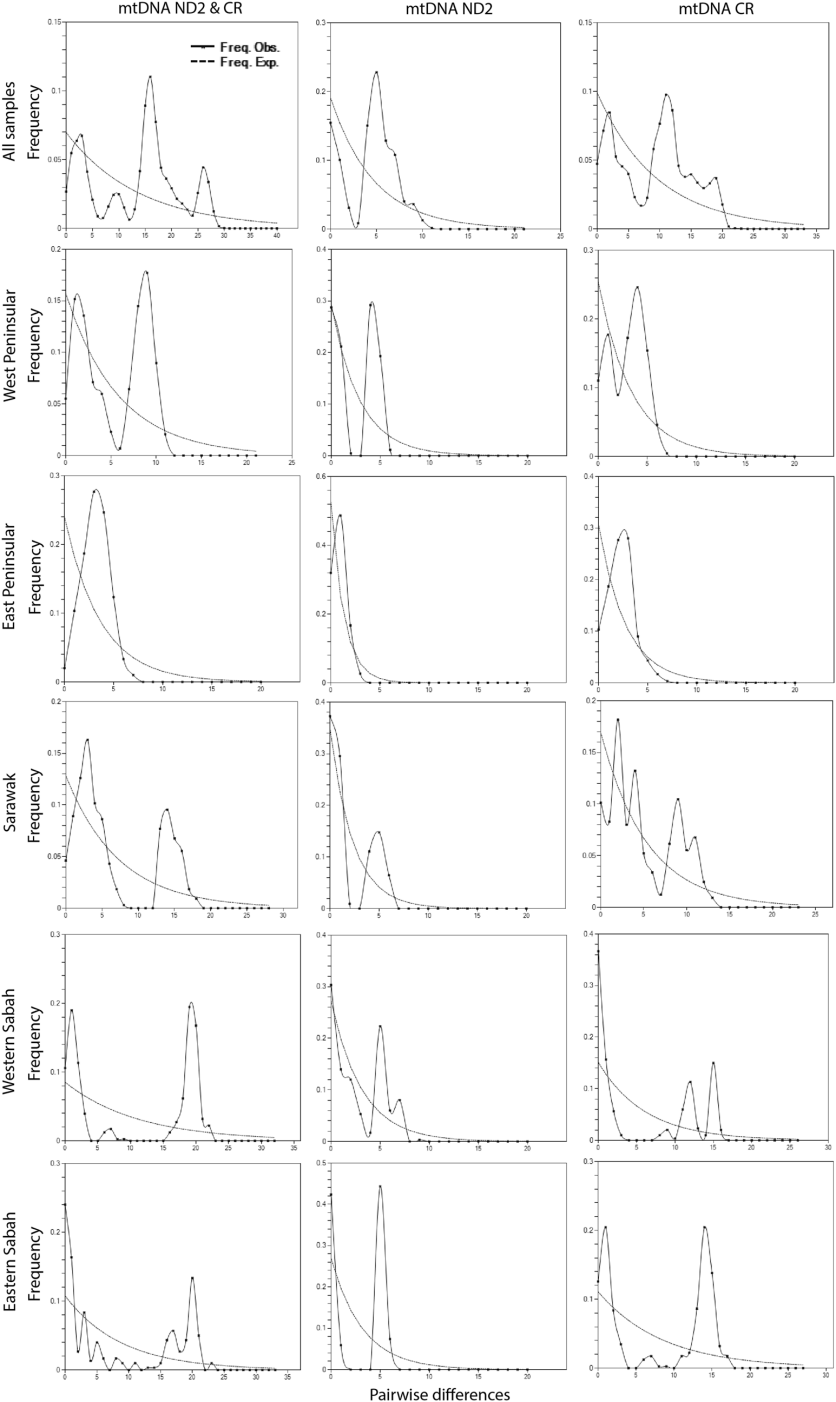
Historical demography of *Chiloscyllium punctatum* populations pair-wise mismatch distribution analyses inferred from individual and combined mtDNA ND2 and control region markers. The distributions are shown for total samples and all major study areas.

The Tajima’s D tests were all not significantly different from zero which suggested no evidence of departure from neutrality (Table 1). On the other hand, Fu's Fs which is more sensitive to recent population expansion^57^ suggested some evidence for population expansion with significantly negative values for both CR and concatenated markers for WP, EP and SR populations (Table 1). The mismatch distribution analysis for the individual and concatenated markers showed consistent results: across all sampling areas; only EP had smooth and unimodal distributions that closely fitted the expected distribution under the sudden expansion model (Fig. 1). The distribution for total samples and all other sampling areas demonstrated a multimodal distribution that is typically attributed to populations in demographic equilibrium.

**Figure 2:**
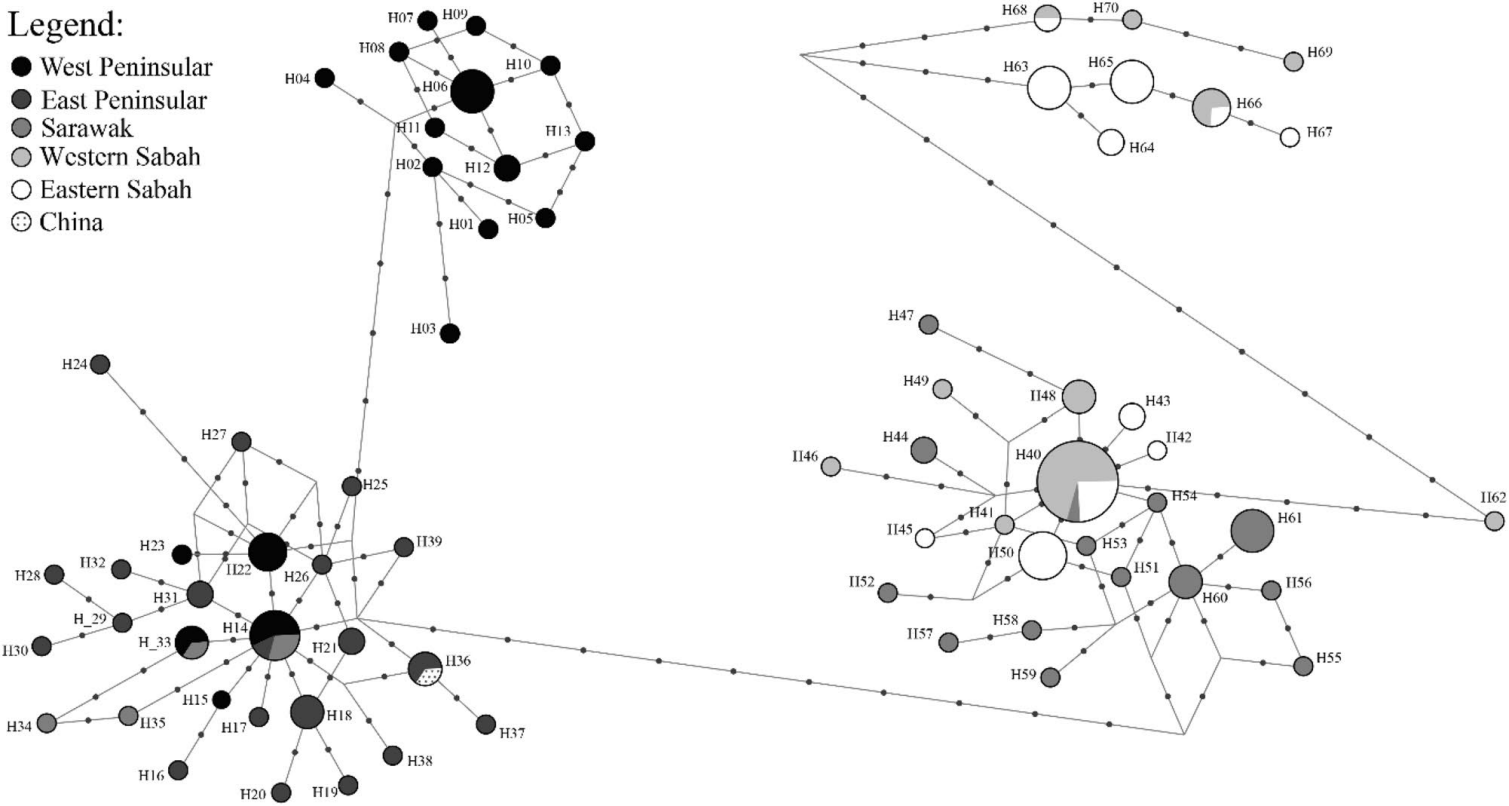
Haplotype network for *Chiloscyllium punctatum* (n = 135). A sequence from an individual sampled from China from NCBI Genbank (JQ082337) is included for comparison. Circle size is proportional to number of samples sharing the haplotypes. Dots along the lines represent number of mutations between the connected haplotypes.

The haplotype network showed that most haplotypes were found almost exclusively within an area (Fig. 2). Only five haplotypes were found across multiple sampling areas, i.e. H14 was found in EP, WP and SR; H33 in EP and SR; H40 in SR, WS and ES; and both H66 and H68 in WS and ES (Supplementary Table S3). In general, four major haplotype clusters were found among the samples—one with haplotypes found only in WP, one with haplotypes found mostly in EP; one with mixed haplotypes from SR, WS and ES; and one with haplotypes from both WS and ES. The single sample obtained from GenBank (sampled in China) clustered together with haplotypes from EP. For samples from Sabah (WS and ES), four sub-clusters separated by a minimum of five mutations were observed. This includes one large cluster with nine haplotypes (seen in 18 individuals from WS, 14 from ES and one from SR) and three minor clusters each with five (three from WS and 14 from ES), three (three from WS and one from ES) and one (one from WS) haplotypes.

**Figure 3:**
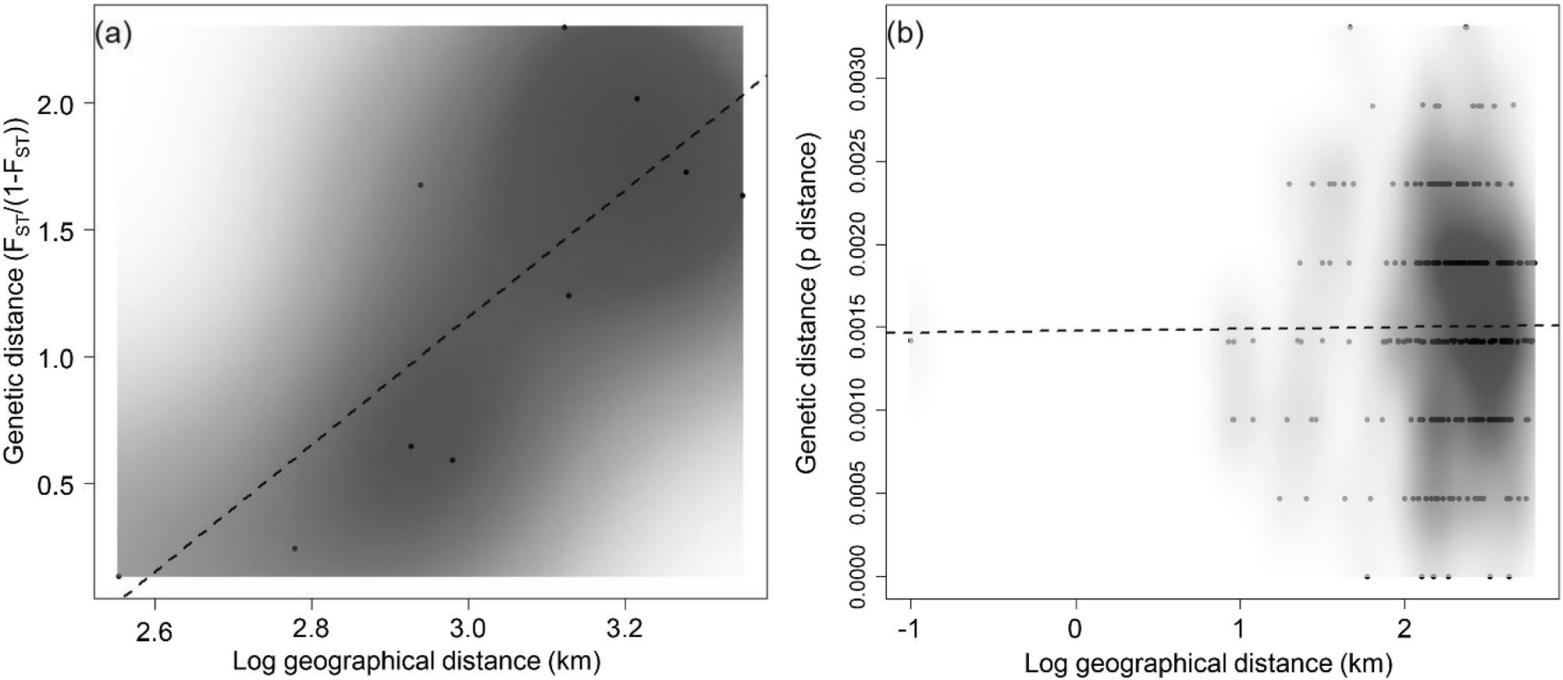
Scatterplot of pairwise genetic distance and log10 of geographical distance (km) among (**a**) the five sampling areas of West Peninsular, East Peninsular, Sarawak, Western Sabah and Eastern Sabah, (**b**) among EP individuals.

Overall, there was a weak but significant support for IBD between areas (r^2^ = 0.62, p < 0.05), with a broad range of pairwise genetic distances at all geographical distance concentrated mostly along the regression line (Fig. 3a). Pairwise comparison supported the high genetic differentiation (p < 0.05) found between all pairs of sampling areas, including weak significance between the two areas within Sabah (WS and ES) (Table 2). Results from the hierarchical AMOVA demonstrated 40–50% of genetic variation was attributed to differences among sampling areas (Table 3). When testing for genetic partitioning under various biogeographical barrier combinations, significant hierarchical partitioning of genetic variation at all levels for both markers was observed when only South China Sea was considered as a genetic barrier (the B2 only scenario). Under this scenario, 46.2% of genetic variation was partitioned among population groups, 15.73% among populations within group, and 38.07% within populations.

**Table 2:** Pairwise F_ST_ (lower diagonal) among sampling sites and respective p-values (upper diagonal) according to markers.

	West Peninsular	East Peninsular	Sarawak	Western Sabah	Eastern Sabah
**CR**					
West Peninsular	–	***	***	***	***
East Peninsular	0.3319	–	***	***	***
Sarawak	0.5366	0.6110	–	***	***
Western Sabah	0.6465	0.7029	0.2218	–	*
Eastern Sabah	0.6111	0.6576	0.3763	0.1254	–
**ND2**					
West Peninsular	–	***	***	***	***
East Peninsular	0.4658	–	***	***	***
Sarawak	0.5819	0.6621	–	***	***
Western Sabah	0.6097	0.6836	0.1258	–	*
Eastern Sabah	0.6384	0.6975	0.3603	0.0920	–
**ND2CR**					
West Peninsular	–	***	***	***	***
East Peninsular	0.3934	–	***	***	***
Sarawak	0.5554	0.6280	–	***	***
Western Sabah	0.6347	0.6980	0.1957	–	*
Eastern Sabah	0.6222	0.6700	0.3736	0.1164	–

* and *** represent statistical significance at p < 0.05 and p < 0.001, respectively.

**Table 3:** AMOVA analysis comparing the genetic variation grouped by various biogeographical barrier (B1, B2 and B3) among genetic markers.

Source of variation	CR					ND2					ND2CR				
	DF	SS	VC	PV		DF	SS	VC	PV		DF	SS	VC	PV	
**No group**															
Among populations	5	307.992	2.714***	52.84	Φ_ST_ = 0.528	5	148.056	1.309***	54.83	Φ_ST_ = 0.548	5	461.087	4.068***	53.61	Φ_ST_ = 0.536
Within populations	130	314.881	2.422	47.16		130	140.115	1.078	45.17		130	457.656	3.520	46.39	
Total	135	622.873	5.136			135	288.171	2.386			135	918.743	7.588		
**Four groups** **(B1, B2 and B3) (WP, EP, SR, WS-ES)**															
Among groups	4	290.966	2.405^n.s^	44.78	Φ_SC_ = 0.183	4	143.015	1.287^n.s^	51.22	Φ_SC_ = 0.120	4	438.849	3.736^n.s^	46.97	Φ_SC_ = 0.165
Among populations within groups	1	17.026	0.544*	10.13	Φ_ST_ = 0.549	1	5.041	0.148*	5.87	Φ_ST_ = 0.571	1	22.237	0.697*	8.76	Φ_ST_ = 0.557
Within populations	130	314.881	2.422***	45.10	Φ_CT_ = 0.448	130	140.115	1.078***	42.90	Φ_CT_ = 0.512	130	457.656	3.520***	44.26	Φ_CT_ = 0.470
Total	135	622.873	5.371			135	288.171	2.512			135	918.743	7.953		
**Three groups (B2 and B3) (WP-EP, SR, WS-ES)**															
Among groups	3	271.678	2.662*	46.98	Φ_SC_ = 0.194	3	121.478	1.070^n.s^	41.17	Φ_SC_ = 0.295	3	397.716	3.779^n.s^	45.31	Φ_SC_ = 0.228
Among populations within groups	2	36.314	0.581***	10.26	Φ_ST_ = 0.572	2	26.578	0.451***	17.36	Φ_ST_ = 0.585	2	63.370	1.041***	12.48	Φ_ST_ = 0.578
Within populations	130	314.881	2.422***	42.75	Φ_CT_ = 0.470	130	140.115	1.078***	41.47	Φ_CT_ = 0.412	130	457.656	3.520***	42.21	Φ_CT_ = 0.453
Total	135	622.873	5.666			135	288.171	2.599			135	918.743	8.340		
**Three groups (B1 and B2) (WP, EP, SR-WS-ES)**															
Among groups	3	242.383	2.205^n.s^	38.23	Φ_SC_ = 0.320	3	128.071	1.366*	49.15	Φ_SC_ = 0.237	3	374.596	3.612^n.s^	41.88	Φ_SC_ = 0.298
Among populations within groups	2	65.608	1.142***	19.79	Φ_ST_ = 0.580	2	19.985	0.335***	12.06	Φ_ST_ = 0.612	2	86.491	1.493***	17.31	Φ_ST_ = 0.592
Within populations	130	314.881	2.422***	41.98	Φ_CT_ = 0.382	130	140.115	1.078***	38.79	Φ_CT_ = 0.492	130	457.656	3.520***	40.82	Φ_CT_ = 0.419
Total	135	622.873	5.769			135	288.171	2.779			135	918.743	8.625		
**Two groups (B2 only) (WP-EP, SR-WS-ES)**															
Among groups	2	223.095	2.860*	45.79	Φ_SC_ = 0.285	2	106.534	1.362^n.s^	46.71	Φ_SC_ = 0.306	2	333.463	4.272**	46.20	Φ_SC_ = 0.292
Among populations within groups	3	84.896	0.964***	15.44	Φ_ST_ = 0.612	3	41.522	0.476***	16.32	Φ_ST_ = 0.630	3	127.624	1.454***	15.73	Φ_ST_ = 0.619
Within populations	130	314.881	2.422***	38.78	Φ_CT_ = 0.458	130	140.115	1.078***	36.97	Φ_CT_ = 0.467	130	457.656	3.520***	38.07	Φ_CT_ = 0.462
Total	135	622.873	6.247			135	288.171	2.915			135	918.743	9.247		

* and *** represent statistical significance at p < 0.05 and p < 0.001, respectively.

**Figure 4:**
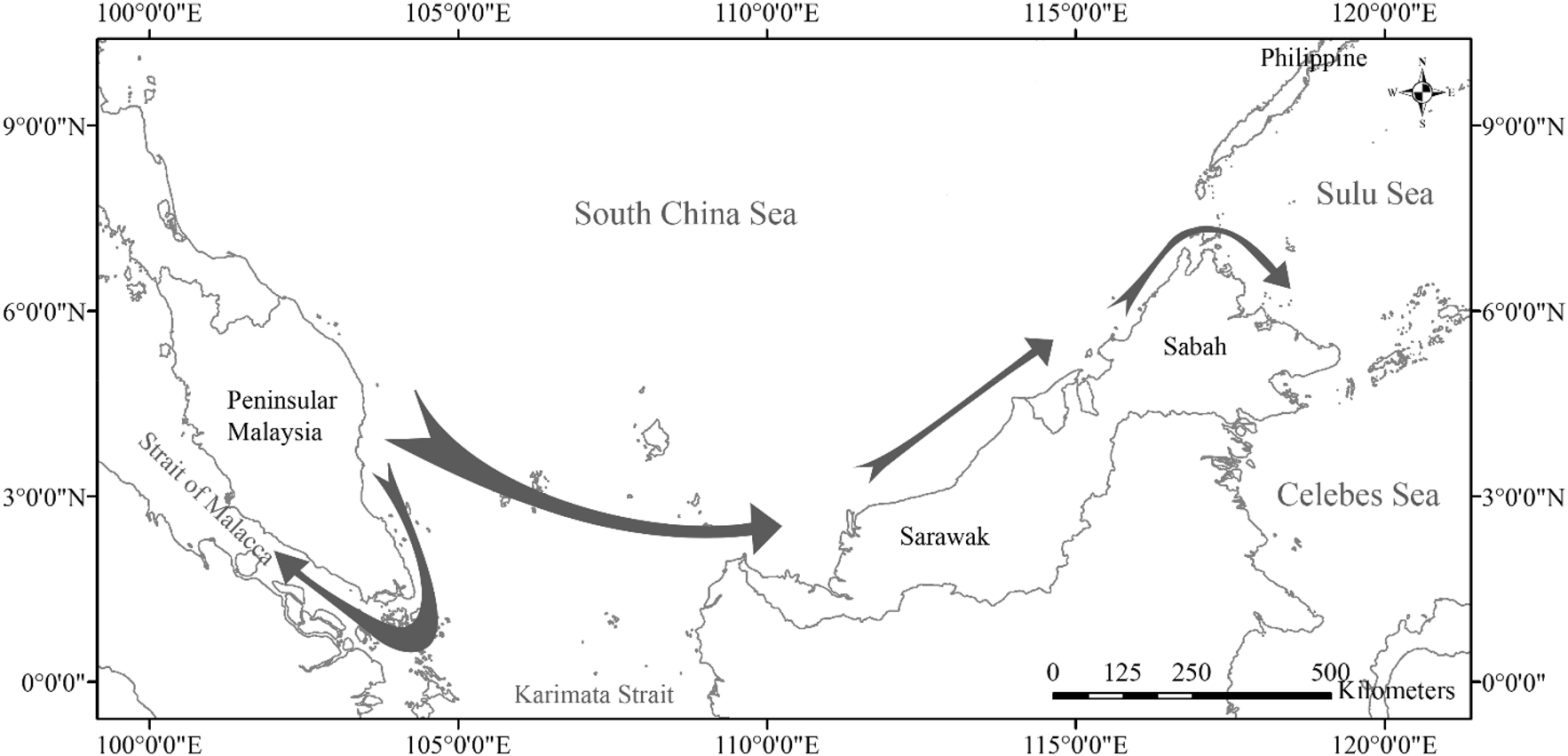
Migration route of *Chiloscyllium punctatum* in Malaysian waters. Direction of the arrows was based on Migrate-n model testing result. Width of arrow indicates the probability of the migration direction at 50,000 steps model (see Table 4 for actual values).

Migrate-n analyses with 10,000 steps and 50,000 steps reached convergence and were both in agreement on the direction of migration between pairs of sampling areas across all pairwise comparisons, with exception of the SR–WS pair (Table 4). An additional analysis using 100,000 steps concurred with the results using 50,000 steps for the SR–WS pair. The best models showed that gene flow occurred from EP to WP and from EP to SR. Specifically for *C. punctatum* sampled from Borneo, model results showed greatest support for gene flow directionality from SR to WS and from WS to ES (Table 4, Fig. 4, Supplementary Tables S4, S5).

**Table 4:** Migrate-n model testing for ND2CR among the sampling area under 10,000 and 50,000 steps. The model with highest support is highlighted in bold.

Area 1	Area 2	Model	10,000 steps			50,000 steps		
			Bezier log marginal-likelihood	Model choice	Probability	Bezier log marginal-likelihood	Model choice	Probability
West Peninsular	East Peninsular	1 ↔ 2	− 3203.31	3	0.001	− 3201.53	2	0.005
		1 → 2	− 3202.68	2	0.001	− 3202.95	3	0.001
		**1 ← 2**	− **3195.81**	**1**	**0.998**	− **3196.33**	**1**	**0.993**
		1 ≠ 2	− 3212.34	4	0.000	− 3213.29	4	0.000
East Peninsular	Sarawak	1 ↔ 2	− 3286.15	3	0.001	− 3284.50	3	0.002
		**1 → 2**	− **3279.11**	**1**	**0.966**	− **3278.33**	**1**	**0.957**
		1 ← 2	− 3282.49	2	0.033	− 3281.97	2	0.025
		1 ≠ 2	− 3306.75	4	0.000	− 3306.72	4	0.000
Sarawak	Western Sabah	1 ↔ 2	− 3319.68	4	0.017	− 3321.82	4	0.007
		**1 → 2**	− 3317.22	3	0.194	− **3317.31**	**1**	**0.642**
		1 ← 2	− **3316.12**	**1**	**0.582**	− 3318.12	2	0.286
		1 ≠ 2	− 3317.15	2	0.208	− 3319.59	3	0.066
Sarawak	Eastern Sabah	1 ↔ 2	− 3359.40	3	0.004	− 3360.90	3	0.001
		**1 → 2**	− **3354.00**	**1**	**0.990**	− **3354.27**	**1**	**0.993**
		1 ← 2	− 3359.15	2	0.006	− 3359.38	2	0.006
		1 ≠ 2	− 3376.44	4	0.000	− 3374.83	4	0.000
Western Sabah	Eastern Sabah	1 ↔ 2	− 3152.67	4	0.041	− 3151.58	2	0.259
		**1 → 2**	− **3149.85**	**1**	**0.684**	− **3150.61**	**1**	**0.684**
		1 ← 2	− 3150.96	2	0.225	− 3153.34	3	0.045
		1 ≠ 2	− 3152.46	3	0.050	− 3156.54	4	0.002

**Figure 5:**
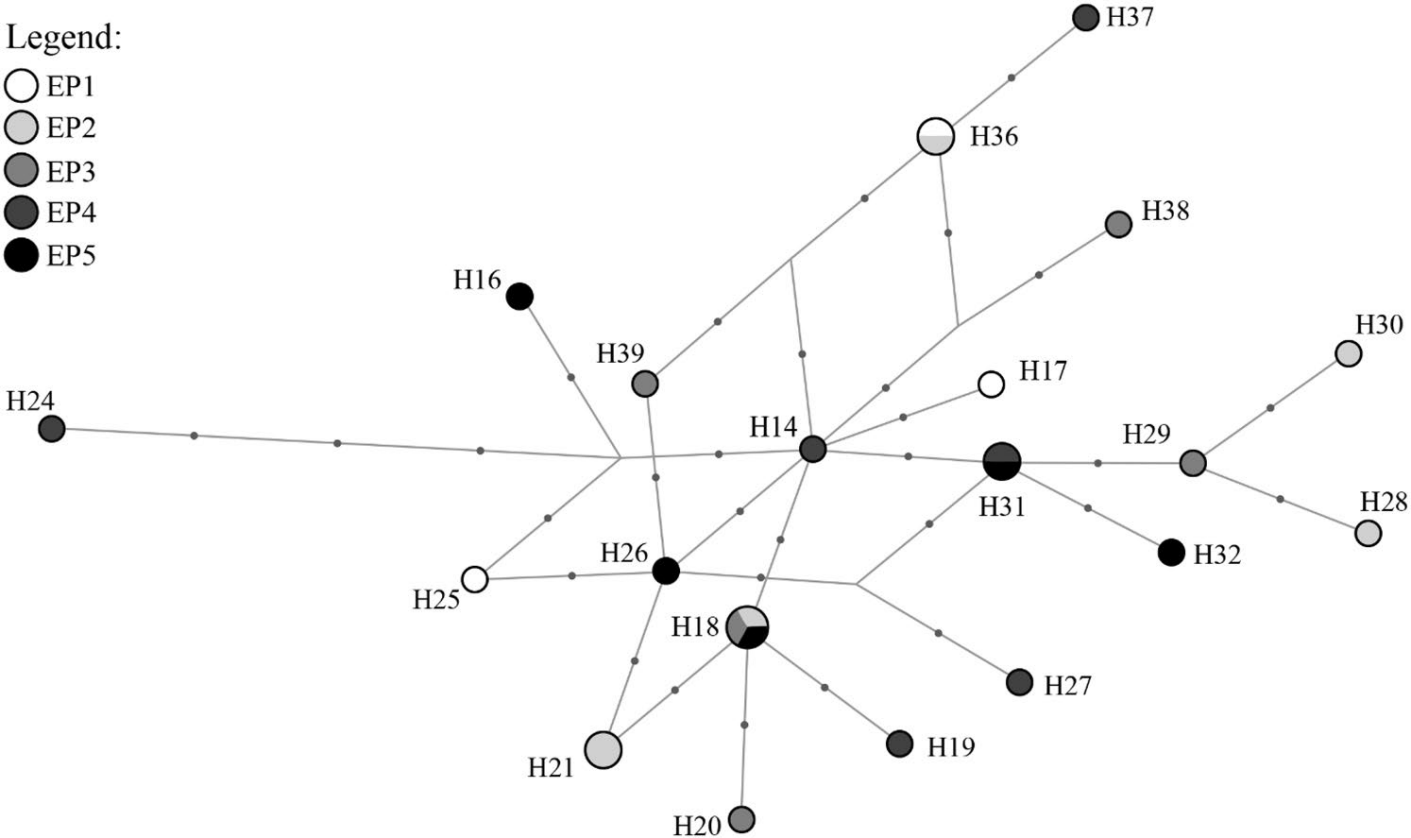
Haplotype network for the five subpopulations of *Chiloscyllium punctatum* from East Peninsular (n = 25). Dots along the line represent number of mutations between the connected haplotypes.

To determine the genetic structure and dispersal pattern at a more local scale, we assessed the level of genetic differentiation, haplotype network and the presence of Isolation-By-Distance (IBD) among the a priori subpopulations along the coastline of EP. No significant genetic differentiation was found among subpopulations (p > 0.05) (Table 5). Congruent with this finding, the EP haplotype network showed no clear patterns of geographical segregation of haplotypes (Fig. 5). For instance, H18 was shared among EP2, EP3, and EP5; H25 from EP1 was one mutation away from H26 from EP5 (Supplementary Tables S1 and S2). Furthermore, IBD analysis showed no correlation between genetic and geographical distances for these subpopulations (r^2^ = 4.73 × 10^–5^, p > 0.05) (Fig. 3b).

**Table 5:** Pairwise F_ST_ (lower diagonal) among subpopulations from the east coast of Peninsular Malaysia and respective significance p-values (upper diagonal) according to markers.

	EP1	EP2	EP3	EP4	EP5
**CR**					
EP1	–	ns	ns	ns	ns
EP2	0.1518	–	ns	ns	ns
EP3	− 0.0934	− 0.2035	–	ns	ns
EP4	− 0.0666	− 0.0302	− 0.0447	–	ns
EP5	− 0.0235	− 0.0477	− 0.0574	− 0.0725	–
**ND2**					
EP1	–	ns	ns	ns	ns
EP2	− 0.0740	–	ns	ns	ns
EP3	− 0.0066	− 0.1661	–	ns	ns
EP4	− 0.0589	0.0211	0.0463	–	ns
EP5	− 0.0936	− 0.0199	0.0277	− 0.1502	–
**ND2CR**					
EP1	–	ns	ns	ns	ns
EP2	− 0.0185	–	ns	ns	ns
EP3	− 0.0621	− 0.1897	–	ns	ns
EP4	− 0.0648	− 0.0154	− 0.0225	–	ns
EP5	− 0.0401	− 0.0392	− 0.0357	− 0.0850	–

*represent significant difference at p < 0.05 after FDR correction.

## Discussion

Using mitochondrial DNA markers for the bamboo shark *C. punctatum,* we were able to show (i) strong genetic differentiation with little present day mixing between the five areas (populations) sampled, (ii) high intra-population genetic diversity with unique haplotypes, (iii) significant correlation between genetic differentiation and geographical distance coupled with detectable presence of fine scale geographical barriers (i.e. the South China Sea), (iv) historical directional gene flow from EP bifurcating towards WP and towards Borneo (from the western end of SR to Sabah), and (v) no detectable genetic differentiation along the EP coastline. The high genetic structure observed in *C. punctatum* within the central area of its distribution range is also consistent with their strong coastal association and life history strategies. We discuss these results in relation to biological and geographical barriers that shape the animal’s phylogeography and implications for fishery management of this small-sized shark.

### Genetic diversity

The large number of unique mtDNA haplotypes in *C. punctatum* found within a small region had exceeded those reported for other elasmobranchs sampled across broader geographical range, e.g. 26 haplotypes in *Negaprion brevirostris*^58^, 39 in *Carcharhinus sorrah*^56^, and 12 in *Ginglymostoma cirratum*^17^. The exception is a report of 67 haplotypes found in *C. plumbeus*^59^. Analysis using cumulative haplotype curves suggested that there are many haplotypes remaining to be discovered for *C. punctatum* (Supplementary Figure S1). Compared to similar sized species of sharks^60^, haplotype diversity of *C. punctatum* is relatively high while the nucleotide diversity is comparable. High number of haplotypes seen in *C. punctatum* could be partially explained by the haplotype genealogy^61^ and the relationship between life history strategies and mutation rates^62^. Star-shaped haplotype genealogy and high haplotype diversity with low nucleotide diversity is a typical genetic signature of recent population expansion, especially when a new habitat became available^61^. Evidence from this study showed some support for selective directional population expansion for *C. punctatum* although the demographic history may not be shared similarly for all the populations in Malaysia (discussed further below).

The high haplotype diversity and directional population expansion detected in our *C. punctatum* samples underscores the need for careful fisheries management to maintain the natural diversity of this highly exploited species and the role of the Malaysian stock as a source population. To date, there is no study that directly assess the relationship between overfishing and genetic diversity in shark and ray species^5^. Overharvesting of marine fish species has been shown to result in reduced mean body size, faster growth rate, and earlier sexual maturity^63^, as well as lower genetic diversity, especially allelic richness^64^. On the other hand, the level of genetic diversity in fishes was surprisingly not found to be lower for threatened species relative to non-threatened ones^65^. Our findings thus provide an important baseline data for key genetic diversity metrics that can be used as indicators of population health and overall conservation status of *C. punctatum*.

### Genetic structure and phylogeography

The lack of genetic structure along the coastline of EP, absence of IBD and sharing of haplotype H18 among EP subpopulations suggests high gene flow and that mixing of *C. punctatum* genetic information could occur as far as 400 km, the longest distance between two known catch locations of the trawl survey conducted. The coastwide genetic mixing can either be facilitated by adult movement or dispersal of egg cases. No mobility study had been conducted on *C. punctatum* or other congeners to date. However, a closely related species with similar behaviour, the nurse shark *G. cirratum* (total length, TL = 430 cm) is able to travel at least 541 km^66^ and genetic studies on the species indicated possibility of longer distance travel along a ~ 5000 km coastline^17^. Another continental shelf species, South African catshark *Poroderma africanum* (TL = 101 cm) could travel as far as 1964 km^66^.

Since coastal depth is probably one major restriction for adult *C. punctatum* movement, ancient and present day bathymetry maps of this region suggest that the species should be able to travel along the shallow waters of coastal EP connected to WP along the Johore Strait (also known as the Tebrau Strait, with maximum depth of 16.9 m) or Singapore Strait and through the Karimata Strait to the Borneo side^67–69^. However significant genetic differentiation and weak support for IBD in *C. punctatum* across the five sampling areas indicates that benthic seascape features may form substantial barriers for adult shark movement across EP-WP and EP-SR. The barrier between EP and WP could result from intensive anthropogenic activities at the Johor and Singapore Straits. These straits, though only approximately 50 km in length, are well known for major reclamation and busy shipping traffic since 1819^70^. Furthermore, industrial activities within the Straits are likely to impact benthic habitat availability and deter adult movement between either coasts of Peninsular Malaysia. The Karimata Strait between EP and SR had been reported to have strong unpredictable currents^71,72^, thus potentially limiting adult movement across the Strait between EP and SR at the western end of Borneo. Study on movement and benthic habitat use for *C. punctatum* will be useful to corroborate the role of distance and bottom features that shape present day genetic structure.

The westward gene flow directionality observed from EP to WP and eastward flow from EP to Borneo (Fig. 4) could be partially explained by contemporary surface ocean currents. During the north-east monsoon, ocean current flows from EP towards WP and towards SR at about 0.3–0.5 m/s^73^; this would be most likely consistent with a pelagic stage dispersal. Egg cases for various elasmobranch species had been recorded to have holding abilities, adhesive fibre in the case of *C. punctatum*, to prevent drifting, exposure to predation or damage by wave and tidal action^74–76^. However, ROV video footage capturing movement of egg cases along with water currents lend support for the possibility of egg case dispersal as a mode of limited population connectivity across these areas^77^. The gene flow directionality implies that population in EP could act as a source for populations in Borneo and in WP.

Unexpectedly, significant genetic differentiation in *C. punctatum* could also be observed along the northern Borneo coastline of about 385 km. Detection of a biogeographical barrier between Sarawak and Sabah suggests that benthic seascape features coupled with differing water bodies (South China Sea and Sulu Sulawesi Sea) are important in shaping the genetic structure for *C. punctatum* of Borneo and possible other similar sharks. This physical delineation is sufficiently significant that *Scoliodon macrorhynchos*, a small sized spadenose shark, could be found along the coastline of the Sarawak Borneo but not of Sabah^Then, unpublished data^. In relation to the weak genetic structuring observed between eastern and western Sabah, further investigation is warranted. Discussions with local fishers and other researchers suggest that sharks landed in Sabah, especially by trawlers, could have been caught from the waters of the Philippines (from the Sulu-Sulawesi waters) Thus, the genetically differentiated Sabah populations and their transboundary relationship with putative populations from the Philippines should be jointly considered.

The genetic differentiation and possible directional population expansion observed in this study could reflect historical vicariance events in the Sunda Shelf region. Other marine organisms in the Indo-Malaya archipelago showed genetic separation between the east and west peninsular populations. These range from smaller and less mobile species such as gastropods *Nerita albicilla*^78^ and *Echinolittorina* spp.^49^, echinoderm *Diadema setosum*^54^, small bony fishes (mudskipper *Periophthalmus argentilineatus*, and reef fish *Centropyge loriculus*)^50,54^, to larger and more mobile species like wedgefish *Rhynchobatus australiae*^55^. The congruency between our data and these past studies indicated the importance of the Sunda Shelf barrier in shaping the phylogeography of marine species in this region.

Overall, the genetic patterns and differentiation observed for *C. punctatum* inferred from the mitochondrial DNA loci would reflect signatures that occurred over historical time scale rather than during contemporary ecological time scale. However, an additional possibility to the strong genetic structure observed can be due to behavioural traits, for instance philopatry or site fidelity which has been shown for diverse species of sharks^79^; thus this cannot be ruled out for the strongly coastal-associated *C. punctatum*. The joint use of nuclear and mitochondrial markers will be important to discern whether the observed patterns of genetic structure in *C. punctatum* could be attributed to philopatric tendencies or due to the presence of biogeographic barriers, or both.

### Fisheries management implications

*Chiloscyllium punctatum* experiences high fishing pressure despite being caught primarily as non-target species (bycatch). In 2017 alone, we estimated that approximately 670,000 individuals were caught and landed at the four major shark landing states in Malaysia (namely Perak, Pahang, Sarawak, and Sabah), based on estimated landings of 750 metric tonnes of *C. punctatum* and average weight of *C. punctatum* landed in trawl fisheries of 1.12 kg^31,80^^, Lim unpublished data^. The national demersal resources were estimated to have decreased by 80% relative to assumed virgin (unexploited) levels during 1960s and this decrease was attributed to overfishing that had reportedly been ongoing for the past two decades^81^. As this species make up almost half of all sharks caught from trawlers, and almost half of the individuals landed were juveniles, impact of fishing pressure remains a great concern in the long run.

High level of genetic structuring in *C. punctatum* across Malaysian waters coupled with low levels of inter-region connectivity strongly suggests the need to consider separate fishery management stocks and conservation plans for the west coast of Peninsular, east coast of Peninsular and Borneo. These separate populations appeared to rely almost exclusively on self-recruitment (with F_ST_ mostly > 0.25 indicating low migration rate among areas)^35,82^; migrants would also likely find difficulty transmitting their genes locally given the territorial nature of the species. Localized population extinctions may be possible if present fishing pressure is not curbed.

Despite ongoing high fishing pressure on highly differentiated genetic stocks of *C. punctatum*, unique biological traits likely confer considerable resilience to this species. *Chiloscyllium punctatum* has shown the ability for long term sperm storage^83^, is extremely hardy and physiologically adapted to inhabiting environments that undergo cyclical hypoxic conditions (e.g., coral reef flats^84^). However, this is likely not the case for many other coastal-associated shark and ray species within the highly fished waters of Malaysia and other neighbouring countries. In addition to clarifying the genetic structuring of these exploited species, efforts to reduce present fishing effort should be prioritized. The proposed move towards a total ban of bottom trawl fishing within Malaysian waters in the next few years would substantially reduce fishing pressure on sharks and rays.

## Conclusion

We present novel evidence of high genetic structure and diversity of *C. punctatum* within the Indo-Malaya Archipelago. Observed high genetic differentiation between the five sampled areas could be attributed to the Sunda Shelf formation acting as ancient barrier for the animals. This genetic pattern persists in the present day due to their strong coastal shelf association and likely limited adult movement, apart from limited directional gene flow between areas. Presence of contemporary barriers for population connectivity is shaped by both distance and benthic features and limited current-driven egg dispersal. Although present high fishing pressure do not appear to pose immediate concerns for *C. punctatum*, findings of strong genetic structure within the small geographical location considered raises the concern of the population health of other exploited small sized sharks in the area.

## Materials and methods

### Ethical statement

The studied shark species is not legally protected in Malaysia. All sharks were dead when encountered at the landing sites. Permission was obtained from the Department of Fisheries Malaysia (DOFM) for sampling onboard the demersal trawl survey in the east coast of Malaysia, in accordance with the DOFM sampling protocol. Collection permits and sampling protocol for samples in Sabah was approved by the Sabah Biodiversity Council [Access License Reference No: JKM/MBS.1000-2/2 JLD.9 (21–23) and Transfer License Reference No: JKM/MBS. 1000-2/3 JLD.4 (18)].

### Sampling site

**Figure 6:**
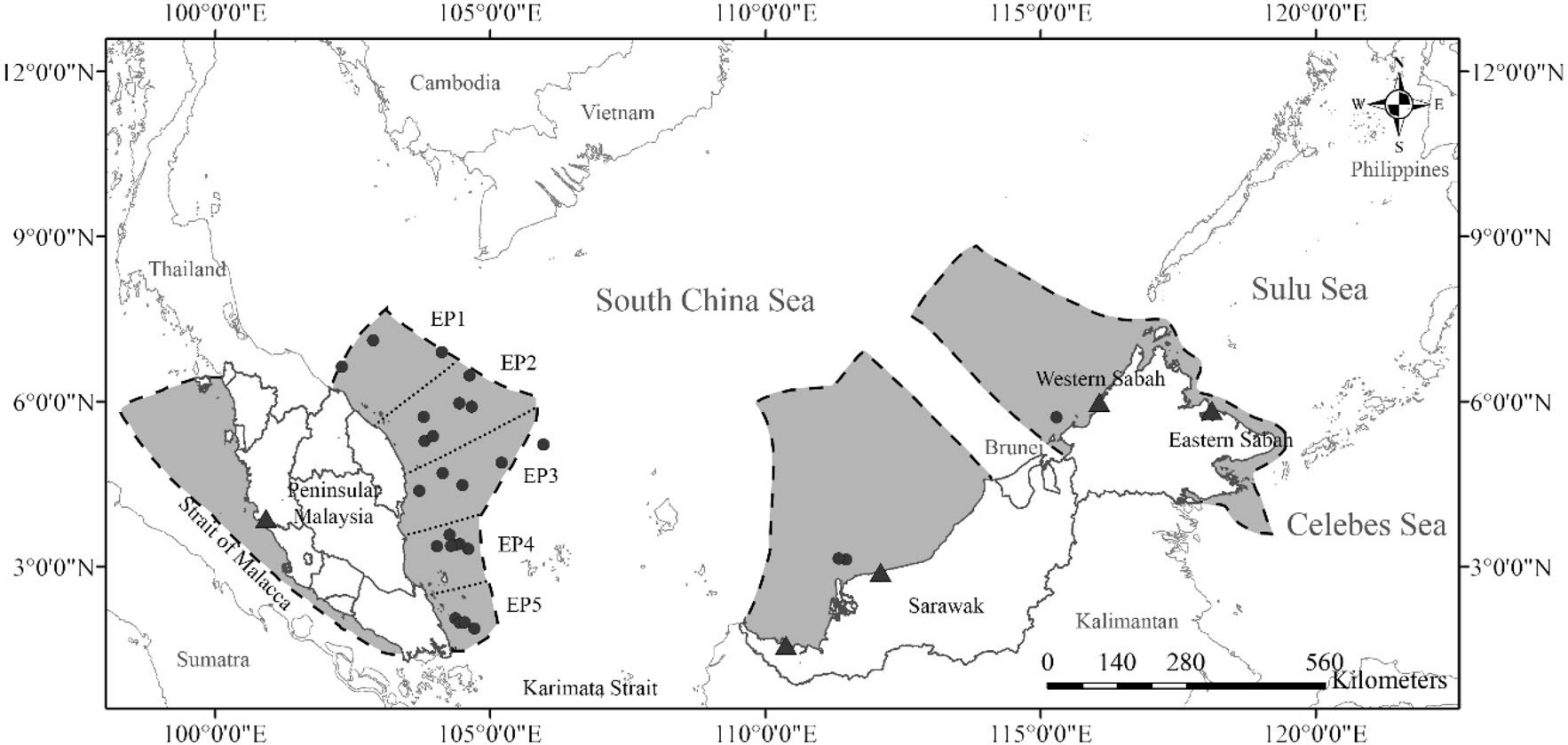
Sampling sites for this study. Triangle = sample collected from landing sites, Circle = samples collected from Department of Fisheries demersal trawl survey, shaded area with dashed line margin denote the Malaysia Economic Exclusive Zone adapted from http://www.marineregions.org, dotted line = subpopulations separation line for East coast of Peninsular Malaysia samples.

Malaysia is located in the centre of the Indo-West Pacific biogeographic region^85^ as well as the distribution range of *C. punctatum*^86^. It is surrounded by three major water bodies namely the Strait of Malacca, southern South China Sea and the Sulu-Celebes Sea (Fig. 6). Sampling was conducted at five major coastal areas: west coast of Peninsular Malaysia (WP), east coast of Peninsular Malaysia (EP), Sarawak (SR), western Sabah (WS) and eastern Sabah (ES) (Fig. 6). The Sabah samples were sub-divided into two areas representing different water bodies; WS faces the South China Sea while ES faces the Sulu-Celebes Sea. These sites were selected to examine the effect of biogeographical barriers to *C. punctatum* populations defined a priori: historical Sunda Shelf Barrier between WP and EP (B1), depth and distance barrier (South China Sea) between EP and SR (B2), and distance barrier between SR and Sabah (B3).

The fin clips of 25–30 individuals per area were collected with a total of 135 individuals (Table 1). With the exception of EP, all other samples were obtained directly at fish landing sites during a 25-month ichthyofaunal survey from September 2015 to September 2017. These animals were caught via commercial fishing operations; the fishing vessels were mainly trawlers except in SR whereby some individuals were caught with gill nets. A maximum of two individuals per fishing vessel were sampled, to prevent over-sampling of maternally-linked individuals that may result in the reduction of potential haplotype diversity^56,87,88^. Samples from EP were obtained through a fisheries-independent demersal trawl survey within the Exclusive Economic Zone (EEZ) organised by the Department of Fisheries Malaysia from July 2015 to July 2016. This invaluable opportunity allowed us to examine finer-scale genetic structure and connectivity of *C. punctatum* along the 724 km-long EP coastline. The EP samples were further divided into five subpopulations (EP1 to EP5) along the coastline (Fig. 6). All tissue samples were preserved in absolute ethanol before subsequent molecular procedures.

### Mitochondrial DNA extraction and analysis

The DNA of each sample was extracted using 10% Chelex resin incubated for 2 min at 60 °C, followed by 25 min at 103 °C (modified from Hyde et al.^89^). Two mitochondrial DNA (mtDNA) markers were employed to analyse the genetic diversity and structure of *C. punctatum*: Control region (CR) and NADH dehydrogenase subunit 2 (ND2). The primer sets used for the targeted regions were CR-F 5′ GAC CTT GTA AGT CGA AGA 3′ and CR-R 5′ TCT TAG CAT CTT CAG TGC 3′ for CR^90^, and Ilem-Mustelus 5'- AAG GAC CAC TTT GAT AGA GT -3' and Asn-Mustelus 5'- AAC GCT TAG CTG TTA ATT AA -3' for ND2^91^. The PCR amplification was performed using a 20 µL reaction mix containing 2 µL of 10 × PCR buffer, 0.5 µL of dNTPs mixture (2.5 mM each), 1 µL of 10 pmol primer (both primers), 1.25 unit of *Taq* DNA polymerase (iNtRON Biotechnology, INC., Korea), 1 µL of 50 pg to 1.0 µg DNA templates, and molecular-grade water. The PCR cycles for both markers comprised of 2 min initial denaturation at 94 °C, followed by 30 cycles of 20 s at 94 °C, 20 s at 52 °C and 1 min at 72 °C, and subsequently a final extension of 5 min at 72 °C. All PCR products were examined using 1% agarose in TAE buffer prior to Sanger sequencing service at Apical Scientific Sdn Bhd (Selangor, Malaysia).

### Data analysis

Sequences of the CR and ND2 regions were reviewed manually, aligned and edited using ClustalW^92^ implemented in Mega version 7 software^93^. All haplotype sequences used for the following analysis were submitted to Genbank database with accession numbers as in Supplementary Table S3. Cumulative haplotype curve using concatenated markers was constructed by randomising the sample order 999 times. The increment of new haplotypes against the number of sequences analysed was plotted using the PRIMER v6 software^94^. This is to observe the increment pattern of haplotype when new sequences was added in the analysis.

An additional sequence from NCBI Genbank (accession number: JQ082337; sampling location: China) that covered both CR and ND2 was included in the analysis to observe potential connection between samples in the South China Sea. Intra and inter-population genetic diversity by individual and concatenated markers (ND2CR) was characterized using D_NA_SP v6 software^95^ with the following indices: the number of haplotypes (*N*_*ha*_), polymorphic sites (*k*) and, haplotype diversity (*ha*) and nucleotide diversity (π). Haplotype or gene diversity is the probability of getting two different alleles from random sampling while nucleotide diversity is the average differences in number of nucleotides per site among DNA sequences^96^. The NETWORK v5.0 software (http://www.fluxus-engineering.com was then used to construct the median-joining haplotype network to visualize the genetic connectivity among the populations.

AMOVA analysis was conducted in ARLEQUIN v3.5.2.2^97^ with 10,000 permutations to identify the hierarchical partitioning of genetic variation under the influence of various combinations of biogeographical barriers stated earlier (B1, B2 and B3). The best model for each marker was selected using jModelTest2; since the best models identified were not implemented in ARLEQUIN, we used the next best available models, i.e., Tamura and Nei correction for CR, Kimura 2 Parameter for ND2 and Kimura 2 Parameter with Gamma 0.656 for ND2CR. Pairwise comparisons of population differentiation using F_ST_ values among (1) subpopulations in EP, and (2) all populations were determined and significant probability values were reported after correction for false discovery rate (FDR)^98^.

Isolation-by-distance (IBD) i.e., correlation between genetic distance and log (base 10) of geographical distances, between populations was calculated and their significance was tested using the Mantel test with 9999 randomizations. Genetic distance between populations was defined as (F_ST_/(1 − F_ST_))^99^. Pairwise geographical distance among populations was estimated as the mean value of the shortest straight-line distance between two points at the sea among the pairs adapting two turning point around southern tip of peninsular to reach WP (west point: 1.1767°N, 103.5197°E; east point: 1.3485°N, 104.3724°E) and one turning point at northern tip of Sabah to ES (7.1231°N, 116.9426°E). For EP, individual-based IBD analysis was performed given that individual catch locations were known and distances for some catch locations within a subpopulation were further than those between subpopulations; uncorrected p-distance calculated in PAUP* 4.0b10 software^100^. The IBD analysis was performed using Adegenet version 2.1.1 package in R version 3.5.1 software^101^.

Historical demographic expansion was inspected using Fu’s^57^ Fs and Tajima’s D tests^102^ of neutrality. Mismatch distribution analysis was subsequently conducted to assess demographic expansion using individual and combined markers in DnaSP v6.10.03^96^. The migration pattern of *C. punctatum* was determined by assessing the migration rate, population size and migration direction using the Bayesian Inference in Migrate-n v3.6.11^103–105^. The analysis was performed on both individual and concatenated markers (ND2CR). Migrate-n allows for the estimation of relative effective population size, θ_Ne_ = (N_e_μ), and asymmetric gene flow, *M* (m/μ), among populations over longer periods of time (> 1000 years)^106^ with the assumption of constant population size, random mating within population, constant mutation rate, constant immigration rate and genetic materials can only exchange through migrants (no population divergence is allowed)^1^. Results from both pairwise testing and haplotype network were used to guide the selection of a priori population pairs for statistical testing and to reduce the number of models considered in Migrate-n. Five population pairs (WP-EP, EP-SR, SR-WS, SR-ES and WS-ES) were subjected to four migration models (1 ↔ 2: bi directional; 1 → 2: unidirectional from population 1 to 2; 1 ← 2: unidirectional from population 2 to 1; 1 ≠ 2: no gene flow) under 10,000 and 50,000 recorded Markov chain Monte Carlo (MCMC) steps; additional analyses using 100,000 steps were done when results did not agree between runs of 10,000 and 50,000 steps. Four-chain heating at temperatures of 1, 1.5, 3 and 1,000,000 was applied to increase the efficiency of the MCMC. Other analysis criteria followed the default settings Modelling protocol and convergence detection followed the guidelines by Beerli et al.^107^. Comparison of models was performed using the Bayes factors based on guidelines by Kass and Raftery^108^.

## Supplementary Information

Supplementary Information.
