# Supplementary material for: Brown banded bamboo shark (Chiloscyllium punctatum) shows high genetic diversity and differentiation in Malaysian waters
Source: Sci Rep. 2021 Jul 21;11:14874. doi: 10.1038/s41598-021-94257-7 (PMC8295251; doi:10.1038/s41598-021-94257-7)
Supplement: Supplementary file 1 — Supplementary Information. [file 41598_2021_94257_MOESM1_ESM.docx]

**Brown banded bamboo shark (*Chiloscyllium punctatum*) shows high genetic diversity and differentiation in Malaysian waters**

Kean Chong Lim^1^, Amy Yee-Hui Then^1,2*^, Alison Kim Shan Wee^3,4^, Ahemad Sade^5^, Richard Rumpet^6^, Kar-Hoe Loh^1*^

**Supplementary information**

**Table S1**

Forty variable sites over 1072 bp of the control region among 70 *Chiloscyllium punctatum* haplotypes

|  | 41 | 103 | 158 | 179 | 181 | 187 | 246 | 248 | 251 | 255 | 274 | 294 | 296 | 326 | 348 | 491 | 537 | 538 | 574 | 608 | 609 | 623 | 645 | 674 | 675 | 676 | 678 | 713 | 719 | 782 | 811 | 828 | 830 | 835 | 844 | 845 | 849 | 865 | 877 | 890 |
| --- | --- | --- | --- | --- | --- | --- | --- | --- | --- | --- | --- | --- | --- | --- | --- | --- | --- | --- | --- | --- | --- | --- | --- | --- | --- | --- | --- | --- | --- | --- | --- | --- | --- | --- | --- | --- | --- | --- | --- | --- |
| H01 | T | C | C | A | T | T | G | C | C | C | C | C | A | G | T | A | G | A | G | G | G | T | T | C | C | T | T | G | A | A | A | A | A | G | G | T | G | G | C | C |
| H02 | . | . | . | . | . | . | . | . | . | . | . | . | . | . | . | . | . | . | . | A | . | . | . | . | . | . | . | . | . | . | . | . | . | . | . | . | . | . | . | . |
| H03 | . | . | T | . | . | . | . | . | . | . | . | . | . | . | . | . | . | . | . | A | . | . | . | . | . | . | . | A | . | . | . | . | . | . | . | . | . | . | . | . |
| H04 | . | T | . | . | . | C | . | . | . | . | . | . | . | . | . | . | . | . | . | A | . | . | . | . | . | . | . | . | . | . | . | . | . | . | . | . | . | . | . | . |
| H05 | . | . | . | . | . | . | . | . | . | . | . | . | . | . | . | . | . | . | . | A | . | . | . | . | . | . | . | . | . | . | . | . | . | . | . | . | . | A | . | . |
| H06 | . | . | . | . | . | C | A | . | . | . | . | . | . | . | . | . | . | . | . | A | . | . | . | . | . | . | . | . | . | . | . | . | . | . | . | . | . | . | . | . |
| H07 | . | . | . | . | . | C | A | . | . | . | . | . | . | . | . | . | . | . | . | A | . | . | . | . | . | . | . | . | T | . | . | . | . | . | . | . | . | . | . | . |
| H08 | . | . | . | . | . | C | A | . | . | . | . | . | . | . | . | . | . | . | . | A | . | . | . | . | . | . | . | . | . | . | G | . | . | . | . | . | . | . | . | . |
| H09 | . | . | . | . | . | C | A | . | . | . | . | . | . | . | . | . | . | . | . | A | . | . | . | . | . | . | . | . | . | . | G | . | . | . | . | . | . | A | . | . |
| H10 | . | . | . | . | . | C | A | . | . | . | . | . | . | . | . | . | . | . | . | A | . | . | . | . | . | . | . | . | . | . | . | . | . | . | . | . | . | A | . | . |
| H11 | . | . | . | . | . | C | A | . | . | . | . | . | . | . | . | . | . | . | . | A | . | . | . | . | . | . | . | . | . | . | G | . | . | . | . | . | . | . | . | . |
| H12 | . | . | . | . | . | C | A | . | . | . | . | . | . | . | . | . | . | . | . | A | . | . | . | . | . | . | . | . | . | . | . | . | . | . | . | . | . | . | . | . |
| H13 | . | . | . | . | . | C | A | . | . | . | . | . | . | . | . | . | . | . | . | A | . | . | . | . | . | . | . | . | . | . | . | . | . | . | . | . | . | A | . | . |
| H14 | . | . | . | . | . | C | . | . | . | . | T | T | . | . | . | . | . | . | . | A | . | . | . | . | . | . | . | . | . | G | . | . | . | . | . | . | . | . | . | . |
| H15 | . | . | . | . | . | C | . | . | . | . | T | T | . | . | . | . | . | . | . | A | . | . | . | . | . | . | . | . | . | G | . | . | . | . | . | . | . | . | . | T |
| H16 | . | . | . | . | . | C | . | . | . | T | T | T | . | . | . | . | . | . | . | A | . | . | . | . | . | . | . | . | . | G | . | . | . | . | . | . | . | . | . | T |
| H17 | . | . | . | . | . | C | . | . | . | . | T | T | . | . | . | . | . | . | . | A | . | . | . | . | . | . | . | . | . | G | . | . | . | . | . | . | . | . | . | . |
| H18 | C | . | . | . | . | C | . | . | . | . | T | T | . | . | . | . | . | . | . | A | . | . | . | . | . | . | . | . | . | G | . | . | . | . | . | . | . | . | . | . |
| H19 | C | . | . | . | . | C | . | . | . | . | T | T | . | . | . | . | . | . | . | A | . | . | . | . | . | . | . | . | . | G | . | . | . | . | . | . | . | . | . | . |
| H20 | C | . | . | . | . | C | . | . | . | . | T | T | . | . | . | . | . | . | . | A | . | . | . | . | . | . | . | . | . | G | . | . | . | . | . | . | . | . | . | . |
| H21 | C | . | . | . | . | C | . | . | . | . | T | T | . | . | . | . | . | . | . | A | . | . | . | . | . | . | . | . | . | G | . | . | . | . | . | . | . | . | . | . |
| H22 | . | . | . | . | . | C | . | . | . | . | T | T | . | . | . | . | . | . | . | A | . | . | . | . | . | . | . | . | . | . | . | . | . | . | . | . | . | . | . | . |
| H23 | . | . | . | . | . | C | . | . | . | . | T | T | . | . | . | . | . | . | . | A | . | . | . | . | . | . | . | . | . | . | . | . | . | . | . | . | . | . | . | . |
| H24 | . | . | . | . | . | C | . | . | . | T | T | T | . | . | . | . | . | . | . | A | . | . | . | . | . | . | . | . | . | . | G | . | G | . | . | . | . | . | . | . |
| H25 | . | . | . | . | . | C | . | . | . | T | T | T | . | . | . | . | . | . | . | A | . | . | . | . | . | . | . | . | . | G | . | . | . | . | . | . | . | . | . | . |
| H26 | . | . | . | . | . | C | . | . | . | . | T | T | . | . | . | . | . | . | . | A | . | . | . | . | . | . | . | . | . | G | . | . | . | . | . | . | . | . | . | . |
| H27 | . | . | . | . | . | C | . | . | . | . | T | T | . | . | . | . | . | . | . | A | . | . | . | . | . | . | . | . | . | . | . | . | . | . | . | . | . | A | . | . |
| H28 | . | . | . | . | . | C | . | . | . | . | T | T | . | . | . | . | . | . | . | A | . | . | . | . | . | . | . | . | . | G | . | . | . | A | . | . | . | A | . | . |
| H29 | . | . | . | . | . | C | . | . | . | . | T | T | . | . | . | . | . | . | . | A | . | . | . | . | . | . | . | . | . | G | . | . | . | . | . | . | . | A | . | . |
| H30 | . | . | . | . | . | C | . | . | . | . | T | T | . | . | . | . | . | . | . | A | . | . | . | . | . | . | . | . | . | G | . | . | . | . | . | . | . | A | . | . |
| H31 | . | . | . | . | . | C | . | . | . | . | T | T | . | . | . | . | . | . | . | A | . | . | . | . | . | . | . | . | . | G | . | . | . | . | . | . | . | A | . | . |
| H32 | . | . | . | . | C | C | . | . | . | . | T | T | . | . | . | . | . | . | . | A | . | . | . | . | . | . | . | . | . | G | . | . | . | . | . | . | . | A | . | . |
| H33 | . | . | . | . | . | C | . | . | . | . | T | T | . | . | . | . | . | . | . | A | . | . | . | . | . | . | . | . | . | G | . | . | . | . | . | . | . | . | . | . |
| H34 | . | . | . | . | . | . | . | . | . | . | T | T | . | . | . | . | . | . | . | A | . | . | . | . | . | . | . | . | . | G | . | . | . | . | . | . | . | . | . | . |
| H35 | . | . | . | . | . | . | . | . | . | . | T | T | . | . | . | . | . | . | . | A | . | . | . | . | . | . | . | . | . | G | . | . | . | . | . | . | . | . | . | . |
| H36 | . | . | . | . | . | C | . | . | . | . | . | T | . | . | . | . | . | T | . | A | . | . | . | . | . | . | . | . | . | G | . | . | . | . | . | . | . | . | . | . |
| H37 | . | . | . | . | . | C | . | . | . | . | . | T | . | . | . | . | . | T | . | A | . | . | . | . | . | . | . | . | . | G | . | . | . | . | . | . | . | A | . | . |
| H38 | . | . | . | . | . | C | . | . | . | . | T | T | . | . | . | . | . | T | . | A | . | . | . | . | . | . | . | . | . | G | . | . | . | A | . | . | . | . | . | . |
| H39 | . | . | . | . | . | C | . | . | . | . | . | T | . | . | . | . | . | . | . | A | . | . | . | . | . | . | . | . | . | G | . | . | . | . | . | . | . | . | . | . |
| H40 | . | . | . | . | . | . | A | . | . | . | . | . | G | A | C | . | A | . | A | A | . | . | . | . | . | . | . | . | . | G | . | . | . | . | A | . | . | . | T | . |
| H41 | . | . | . | . | . | . | A | . | . | . | . | . | G | A | C | . | A | . | A | A | . | . | . | . | . | . | . | . | . | G | . | . | . | . | A | . | . | A | T | . |
| H42 | . | . | . | . | . | . | A | . | . | . | . | . | G | A | C | . | A | . | A | A | . | . | . | . | . | . | . | . | . | G | . | . | . | . | A | C | . | . | T | . |
| H43 | . | . | . | . | . | . | A | . | . | . | . | . | G | A | C | . | A | . | A | A | . | . | . | . | . | . | . | . | . | G | . | . | . | . | A | . | . | . | T | . |
| H44 | . | . | . | . | . | . | A | T | . | . | . | . | G | A | C | . | . | . | A | A | . | . | . | . | . | . | . | . | . | G | . | . | . | . | A | . | . | . | T | . |
| H45 | . | . | . | . | . | . | A | . | . | . | . | . | G | A | C | . | . | . | A | A | . | . | . | . | . | . | . | . | . | G | . | . | . | . | A | . | . | A | T | . |
| H46 | . | . | . | . | . | . | A | . | . | . | . | . | G | A | C | . | . | . | A | A | . | . | . | . | . | . | . | . | . | G | . | . | . | . | A | . | . | . | T | . |
| H47 | . | . | . | G | . | . | A | . | T | . | . | . | G | A | C | . | A | . | A | A | . | . | . | . | . | . | . | . | . | G | . | . | . | . | A | . | . | . | T | . |
| H48 | . | . | . | . | . | . | A | . | . | . | . | . | G | A | C | . | A | . | A | A | . | . | . | . | . | . | . | . | . | G | . | . | . | . | A | . | . | . | T | . |
| H49 | . | . | . | . | . | . | A | . | . | . | . | . | G | A | C | . | A | . | A | A | . | . | . | . | . | . | . | . | . | G | . | . | . | . | A | . | . | A | T | . |
| H50 | . | . | . | . | . | . | A | . | . | . | . | . | G | A | C | . | A | . | A | A | . | . | . | . | . | . | . | . | . | G | . | . | . | . | A | . | . | . | . | . |
| H51 | . | . | . | . | . | . | A | . | . | . | . | . | . | A | C | . | A | . | A | A | . | . | . | . | . | . | . | . | . | G | . | . | . | . | A | . | . | . | . | . |
| H52 | . | . | . | . | . | . | A | . | T | . | . | . | G | A | C | . | A | . | A | A | . | . | . | . | . | . | . | . | . | G | . | . | . | . | A | . | . | A | . | . |
| H53 | . | . | . | . | . | . | A | . | . | . | . | . | . | A | C | . | A | . | A | A | . | . | . | . | . | . | . | . | . | G | . | . | . | . | A | . | . | A | T | . |
| H54 | . | . | . | . | . | . | A | . | . | . | . | . | . | A | C | . | A | . | A | A | . | . | . | . | . | . | . | . | . | G | . | . | . | . | A | . | . | . | T | . |
| H55 | . | . | . | . | . | . | . | . | . | . | . | . | . | . | C | . | A | . | A | A | . | . | . | . | . | . | . | . | . | G | . | . | . | . | A | . | A | . | T | . |
| H56 | . | . | . | . | . | . | A | . | . | . | . | . | . | . | C | . | A | . | A | A | . | . | . | . | . | . | . | . | . | G | . | . | . | . | A | . | A | . | T | . |
| H57 | . | . | . | . | . | . | A | . | . | . | . | . | . | . | C | G | A | . | A | A | . | . | . | . | . | . | . | . | . | G | . | . | . | . | A | . | A | A | T | . |
| H58 | . | . | . | . | . | . | A | . | . | . | . | . | . | . | C | G | A | . | A | A | . | . | . | . | . | . | . | . | . | G | . | . | . | . | A | . | . | A | T | . |
| H59 | . | . | . | . | . | . | A | . | . | . | . | . | . | . | C | . | A | . | A | A | . | . | . | . | . | . | . | . | . | G | . | . | . | . | A | C | . | A | T | . |
| H60 | . | . | . | . | . | . | A | . | . | . | . | . | . | . | C | . | A | . | A | A | . | . | . | . | . | . | . | . | . | G | . | . | . | . | A | . | . | . | T | . |
| H61 | . | . | . | . | . | . | A | . | . | . | . | . | . | . | C | . | A | . | A | A | . | . | . | . | . | . | . | . | . | G | . | . | . | . | A | . | . | . | T | . |
| H62 | . | . | . | . | . | . | A | . | . | . | . | . | G | A | . | . | A | . | A | A | . | . | . | T | . | . | . | . | . | G | . | . | . | . | A | . | . | . | T | . |
| H63 | . | T | . | . | . | . | A | . | . | . | . | . | G | A | . | . | . | . | A | A | A | C | C | T | T | C | C | . | . | G | . | . | . | T | . | . | . | . | T | T |
| H64 | . | T | . | . | . | . | A | . | . | . | . | . | G | A | . | . | . | . | A | A | A | C | C | T | T | C | C | . | . | G | . | . | . | T | . | . | . | A | T | T |
| H65 | . | T | . | . | . | . | A | . | . | T | . | . | G | A | . | . | . | . | A | A | A | C | C | T | T | C | C | . | . | G | . | . | . | T | . | . | . | . | T | T |
| H66 | . | T | . | . | . | . | A | . | . | T | . | . | G | A | . | . | . | . | . | A | A | C | C | T | T | C | C | . | . | G | . | . | . | T | . | . | . | . | T | T |
| H67 | . | T | . | . | . | . | A | . | . | T | . | . | G | A | . | . | . | . | . | A | A | C | C | T | T | C | C | . | . | G | . | . | . | T | . | . | . | A | T | T |
| H68 | . | . | . | . | . | . | . | . | . | . | . | . | G | . | C | . | . | . | A | A | A | . | . | T | T | C | C | . | . | G | . | . | . | T | . | . | . | . | T | T |
| H69 | . | . | . | . | . | . | . | . | . | . | . | . | G | . | C | . | . | . | A | A | A | . | . | T | T | C | C | . | . | G | . | G | . | T | . | . | . | . | T | T |
| H70 | . | . | . | . | . | . | . | . | . | . | . | . | G | . | C | . | . | . | A | A | A | . | . | T | T | C | C | . | . | G | . | G | . | T | . | . | . | . | T | T |

**Table S2**

Twenty-three variable sites over 1044 bp of the ND2 gene among 70 *Chiloscyllium punctatum* haplotypes.

|  | 43 | 180 | 343 | 345 | 483 | 519 | 594 | 612 | 621 | 624 | 663 | 705 | 718 | 759 | 811 | 813 | 826 | 854 | 886 | 906 | 913 | 1001 | 1015 |
| --- | --- | --- | --- | --- | --- | --- | --- | --- | --- | --- | --- | --- | --- | --- | --- | --- | --- | --- | --- | --- | --- | --- | --- |
| H01 | C | C | C | A | T | T | A | T | C | T | A | T | A | C | A | A | A | T | T | A | A | C | C |
| H02 | . | . | . | . | . | . | . | . | . | . | . | . | . | . | . | . | . | . | . | . | . | . | . |
| H03 | . | . | . | . | . | . | . | . | . | . | . | . | . | . | . | . | . | . | . | . | . | . | . |
| H04 | . | . | . | . | . | . | . | . | . | . | . | . | . | . | . | . | . | . | . | . | . | . | . |
| H05 | . | . | . | . | . | . | . | . | . | . | . | . | . | . | . | . | . | . | . | . | . | . | T |
| H06 | . | . | . | . | . | . | . | . | . | . | . | . | . | . | . | . | . | . | . | . | . | . | . |
| H07 | . | . | . | . | . | . | . | . | . | . | . | . | . | . | . | . | . | . | . | . | . | . | . |
| H08 | . | . | . | . | . | . | . | . | . | . | . | . | . | . | . | . | . | . | . | . | . | . | . |
| H09 | . | . | . | . | . | . | . | . | . | . | . | . | . | . | . | . | . | . | . | . | . | . | . |
| H10 | . | . | . | . | . | . | . | . | . | . | . | . | . | . | . | . | . | . | . | . | . | . | . |
| H11 | . | . | . | . | . | . | . | . | . | . | . | . | . | . | . | . | . | . | . | . | . | . | T |
| H12 | . | . | . | . | . | . | . | . | . | . | . | . | . | . | . | . | . | . | . | . | . | . | T |
| H13 | . | . | . | . | . | . | . | . | . | . | . | . | . | . | . | . | . | . | . | . | . | . | T |
| H14 | . | T | . | . | . | . | . | . | T | C | . | . | T | . | . | . | . | . | . | . | . | . | . |
| H15 | . | T | . | . | . | . | . | . | T | C | . | . | T | . | . | . | . | . | . | . | . | . | . |
| H16 | . | T | . | . | . | . | . | . | T | C | . | . | T | . | . | . | . | . | . | . | . | . | . |
| H17 | . | T | . | . | . | . | . | . | T | C | . | . | T | . | . | . | . | . | . | T | . | . | . |
| H18 | . | T | . | . | . | . | . | . | T | C | . | . | T | . | . | . | . | . | . | . | . | . | . |
| H19 | . | T | . | . | . | . | . | . | T | C | . | . | T | . | . | . | . | . | . | . | G | . | . |
| H20 | . | T | . | . | . | . | . | . | T | C | . | . | T | . | C | . | . | . | . | . | . | . | . |
| H21 | . | T | . | . | . | . | . | . | T | C | . | . | T | . | . | C | . | . | . | . | . | . | . |
| H22 | . | T | . | . | . | . | . | . | T | C | . | . | T | . | . | . | . | . | . | . | . | . | . |
| H23 | . | T | . | . | . | . | . | . | T | C | . | . | T | . | C | . | . | . | . | . | . | . | . |
| H24 | . | T | . | . | . | . | . | . | T | C | . | . | T | . | . | . | . | . | . | . | . | . | . |
| H25 | . | T | . | . | . | . | . | . | T | C | . | . | T | . | . | C | . | . | . | . | . | . | . |
| H26 | . | T | . | . | . | . | . | . | T | C | . | . | T | . | . | C | . | . | . | . | . | . | . |
| H27 | . | T | . | . | . | . | . | . | T | C | . | . | T | . | . | C | . | . | . | . | . | . | . |
| H28 | . | T | . | . | . | . | . | . | T | C | . | . | T | . | C | . | . | . | . | . | . | . | . |
| H29 | . | T | . | . | . | . | . | . | T | C | . | . | T | . | C | . | . | . | . | . | . | . | . |
| H30 | . | T | . | G | . | . | . | . | T | C | . | . | T | . | C | . | . | . | . | . | . | . | . |
| H31 | . | T | . | . | . | . | . | . | T | C | . | . | T | . | . | . | . | . | . | . | . | . | . |
| H32 | . | T | . | . | . | . | . | . | T | C | . | . | T | . | . | . | . | . | . | . | . | . | . |
| H33 | . | T | . | . | . | . | . | . | T | C | . | . | T | . | . | . | . | . | . | . | . | . | T |
| H34 | . | T | . | . | . | . | . | . | T | C | . | . | T | . | . | . | . | C | . | . | . | . | T |
| H35 | . | T | . | . | . | . | . | . | T | C | . | . | T | . | . | . | . | C | . | . | . | . | . |
| H36 | . | T | . | . | . | . | . | . | T | C | . | . | T | . | . | . | . | . | . | . | . | . | . |
| H37 | . | T | . | . | . | . | . | . | T | C | . | . | T | . | . | . | . | . | . | . | . | . | . |
| H38 | . | T | . | . | . | . | . | . | T | C | . | . | T | . | . | . | . | . | . | . | . | . | . |
| H39 | . | T | . | . | . | . | . | . | T | C | . | . | T | . | . | C | . | . | . | . | . | . | . |
| H40 | T | T | . | . | . | C | . | . | T | C | . | C | . | . | . | . | . | . | . | . | . | . | . |
| H41 | T | T | . | . | . | C | . | . | T | C | . | C | . | . | . | . | . | . | . | . | . | . | . |
| H42 | T | T | . | . | . | C | . | . | T | C | . | C | . | . | . | . | . | . | . | . | . | . | . |
| H43 | T | T | . | . | . | C | . | . | T | C | . | C | . | . | . | . | . | . | . | . | . | T | . |
| H44 | T | T | . | . | . | C | . | . | T | C | . | C | . | . | . | . | . | . | . | . | . | . | . |
| H45 | T | T | . | . | . | C | . | . | T | C | . | C | . | . | . | . | . | . | . | . | . | . | . |
| H46 | T | T | . | . | C | C | . | . | T | C | . | C | . | T | . | . | . | . | . | . | . | . | . |
| H47 | T | T | . | . | . | C | . | . | T | C | . | C | . | . | . | . | . | . | . | . | . | . | T |
| H48 | T | T | . | . | . | C | . | . | T | C | . | C | . | . | . | . | . | . | . | . | . | . | T |
| H49 | T | T | . | . | . | C | . | . | T | C | . | C | . | . | . | . | C | . | . | . | . | . | T |
| H50 | T | T | . | . | . | C | . | . | T | C | . | C | . | . | . | . | . | . | . | . | . | . | . |
| H51 | T | T | . | . | . | C | . | . | T | C | . | C | . | . | . | . | . | . | . | . | . | . | . |
| H52 | T | T | . | . | . | C | . | . | T | C | . | C | . | . | . | . | . | . | . | . | . | . | . |
| H53 | T | T | . | . | . | C | . | . | T | C | . | C | . | . | . | . | . | . | . | . | . | . | . |
| H54 | T | T | . | . | . | C | . | . | T | C | . | C | . | . | . | . | . | . | . | . | . | . | . |
| H55 | T | T | . | . | . | C | . | . | T | C | . | C | . | . | . | . | . | . | . | . | . | . | . |
| H56 | T | T | . | . | . | C | . | . | T | C | . | C | . | . | . | . | . | . | . | . | . | . | . |
| H57 | T | T | . | . | . | C | . | . | T | C | . | C | . | . | . | . | . | . | . | . | . | . | . |
| H58 | T | T | . | . | . | C | . | . | T | C | . | C | . | . | . | . | . | . | . | . | . | . | . |
| H59 | T | T | . | . | . | C | . | . | T | C | . | C | . | . | . | . | . | . | . | . | . | . | . |
| H60 | T | T | . | . | . | C | . | . | T | C | . | C | . | . | . | . | . | . | . | . | . | . | . |
| H61 | T | T | . | . | . | C | . | . | T | C | . | C | . | . | . | . | . | . | . | . | . | . | T |
| H62 | T | T | T | . | . | C | . | . | T | C | G | . | . | . | . | . | . | . | . | . | . | . | . |
| H63 | T | T | T | . | . | C | C | . | T | C | G | . | . | . | . | . | . | . | C | . | . | . | . |
| H64 | T | T | T | . | . | C | C | . | T | C | G | . | . | . | . | . | . | . | C | . | . | . | . |
| H65 | T | T | T | . | . | C | C | . | T | C | G | . | . | . | . | . | . | . | C | . | . | . | . |
| H66 | T | T | T | . | . | C | C | . | T | C | G | . | . | . | . | . | . | . | C | . | . | . | . |
| H67 | T | T | T | . | . | C | C | . | T | C | G | . | . | . | . | . | . | . | C | . | . | . | . |
| H68 | T | T | T | . | . | C | C | . | T | C | G | . | . | . | . | . | . | . | C | . | . | . | . |
| H69 | T | T | T | . | . | C | C | C | T | C | G | . | . | . | . | . | . | . | C | . | . | . | T |
| H70 | T | T | T | . | . | C | C | . | T | C | G | . | . | . | . | . | . | . | C | . | . | . | . |

**Table S3**

Details for all *Chiloscyllium punctatum* haplotypes obtained in this study. Samples sharing same haplotype is listed in sample code. WP – West Peninsular, E1–E5 – East Peninsular subpopulation 1 to 5, SR- Sarawak, WS – Western Sabah, ES – Eastern Sabah, CR – control region, ND2 – NADH dehydrogenase subunit 2.

| **Haplotype** | **Sample code** | **Gene** | **Accession No.** |
| --- | --- | --- | --- |
| H1 | 2178WP | CR | MT241450 |
|  |  | ND2 | MT241400 |
| H2 | 2864WP | CR | MT241452 |
|  |  | ND2 | MT241400 |
| H3 | 3652WP | CR | MT241451 |
|  |  | ND2 | MT241400 |
| H4 | 4589WP | CR | MT241456 |
|  |  | ND2 | MT241400 |
| H5 | 4738WP | CR | MT241453 |
|  |  | ND2 | MT241401 |
| H6 | 2234WP; 3537WP; 3693WP; 4567WP; 4757WP | CR | MT241455 |
|  |  | ND2 | MT241400 |
| H7 | 3593WP | CR | MT241454 |
|  |  | ND2 | MT241400 |
| H8 | 2402WP | CR | MT241457 |
|  |  | ND2 | MT241400 |
| H9 | 4794WP | CR | MT241458 |
|  |  | ND2 | MT241400 |
| H10 | 4517WP | CR | MT241459 |
|  |  | ND2 | MT241400 |
| H11 | 3604WP | CR | MT241457 |
|  |  | ND2 | MT241401 |
| H12 | 3605WP; 4494WP | CR | MT241455 |
|  |  | ND2 | MT241401 |
| H13 | 3671WP | CR | MT241459 |
|  |  | ND2 | MT241401 |
| H14 | 2204WP; 2980SR; 3405SR; 3505WP; 3680WP; 4039WP; 4426E4 | CR | MT241460 |
|  |  | ND2 | MT241391 |
| H15 | 2217WP | CR | MT241466 |
|  |  | ND2 | MT241391 |
| H16 | 4640E5 | CR | MT241467 |
|  |  | ND2 | MT241391 |
| H17 | 4238E1 | CR | MT241460 |
|  |  | ND2 | MT241394 |
| H18 | 4311E2; 4356E3; 4639E5 | CR | MT241468 |
|  |  | ND2 | MT241391 |
| H19 | 4435E4 | CR | MT241468 |
|  |  | ND2 | MT241395 |
| H20 | 4452E3 | CR | MT241468 |
|  |  | ND2 | MT241392 |
| H21 | 4333E2; 4340E2 | CR | MT241468 |
|  |  | ND2 | MT241396 |
| H22 | 2398WP; 2846WP; 3711WP; 4781WP | CR | MT241469 |
|  |  | ND2 | MT241391 |
| H23 | 4576WP | CR | MT241469 |
|  |  | ND2 | MT241392 |
| H24 | 4415E4 | CR | MT241471 |
|  |  | ND2 | MT241391 |
| H25 | 4332E1 | CR | MT241470 |
|  |  | ND2 | MT241396 |
| H26 | 4645E5 | CR | MT241460 |
|  |  | ND2 | MT241396 |
| H27 | 4424E4 | CR | MT241463 |
|  |  | ND2 | MT241396 |
| H28 | 4312E2 | CR | MT241464 |
|  |  | ND2 | MT241392 |
| H29 | 4448E3 | CR | MT241461 |
|  |  | ND2 | MT241392 |
| H30 | 4307E2 | CR | MT241461 |
|  |  | ND2 | MT241393 |
| H31 | 4427E4; 4635E5 | CR | MT241461 |
|  |  | ND2 | MT241391 |
| H32 | 4630E5 | CR | MT241462 |
|  |  | ND2 | MT241391 |
| H33 | 2826WP; 3062SR; 4031WP | CR | MT241460 |
|  |  | ND2 | MT241397 |
| H34 | 3915SR | CR | MT241472 |
|  |  | ND2 | MT241399 |
| H35 | 3407SR | CR | MT241472 |
|  |  | ND2 | MT241398 |
| H36 | 4202E1; 4292E2 | CR | MT241473 |
|  |  | ND2 | MT241391 |
| H37 | 4412E4 | CR | MT241474 |
|  |  | ND2 | MT241391 |
| H38 | 4346E3 | CR | MT241465 |
|  |  | ND2 | MT241391 |
| H39 | 4404E3 | CR | MT241475 |
|  |  | ND2 | MT241396 |
| H40 | 2458WS; 2460WS; 3067SR; 5058ES; 5063ES; 5076ES; 5077ES; 5120WS; 5121WS; 5124WS; 5126WS; 5127WS; 5156WS; 5157WS; 5162WS; 5211WS; 5241WS | CR | MT241476 |
|  |  | ND2 | MT241402 |
| H41 | 5154WS | CR | MT241486 |
|  |  | ND2 | MT241402 |
| H42 | 5069ES | CR | MT241478 |
|  |  | ND2 | MT241402 |
| H43 | 4797ES; 5107ES | CR | MT241476 |
|  |  | ND2 | MT241403 |
| H44 | 3875SR; 3916SR | CR | MT241487 |
|  |  | ND2 | MT241402 |
| H45 | 5112ES | CR | MT241488 |
|  |  | ND2 | MT241402 |
| H46 | 5133WS | CR | MT241489 |
|  |  | ND2 | MT241404 |
| H47 | 2476SR | CR | MT241481 |
|  |  | ND2 | MT241405 |
| H48 | 5148WS; 5150WS; 5153WS | CR | MT241476 |
|  |  | ND2 | MT241405 |
| H49 | 5129WS | CR | MT241486 |
|  |  | ND2 | MT241406 |
| H50 | 4796ES; 4821ES; 4999ES; 5060ES; 5108ES; 5111ES | CR | MT241479 |
|  |  | ND2 | MT241402 |
| H51 | 2978SR | CR | MT241480 |
|  |  | ND2 | MT241402 |
| H52 | 3893SR | CR | MT241485 |
|  |  | ND2 | MT241402 |
| H53 | 3347SR | CR | MT241490 |
|  |  | ND2 | MT241402 |
| H54 | 3735SR | CR | MT241491 |
|  |  | ND2 | MT241402 |
| H55 | 2478SR | CR | MT241492 |
|  |  | ND2 | MT241402 |
| H56 | 3117SR | CR | MT241493 |
|  |  | ND2 | MT241402 |
| H57 | 3349SR | CR | MT241494 |
|  |  | ND2 | MT241402 |
| H58 | 3874SR | CR | MT241495 |
|  |  | ND2 | MT241402 |
| H59 | 3403SR | CR | MT241496 |
|  |  | ND2 | MT241402 |
| H60 | 3351SR; 3736SR; 3737SR | CR | MT241497 |
|  |  | ND2 | MT241402 |
| H61 | 3068SR; 3409SR; 3844SR; 3894SR; 3976SR | CR | MT241497 |
|  |  | ND2 | MT241405 |
| H62 | 5231WS | CR | MT241477 |
|  |  | ND2 | MT241409 |
| H63 | 4000ES; 4819ES; 4820ES; 4890ES; 5066ES | CR | MT241500 |
|  |  | ND2 | MT241407 |
| H64 | 4823ES; 4870ES | CR | MT241501 |
|  |  | ND2 | MT241407 |
| H65 | 4822ES; 4871ES; 5031ES; 5067ES; 5113ES | CR | MT241502 |
|  |  | ND2 | MT241407 |
| H66 | 5061ES; 5147WS; 5155WS; 5159WS | CR | MT241503 |
|  |  | ND2 | MT241407 |
| H67 | 4891ES | CR | MT241504 |
|  |  | ND2 | MT241407 |
| H68 | 5062ES; 5123WS | CR | MT241499 |
|  |  | ND2 | MT241407 |
| H69 | 5149WS | CR | MT241498 |
|  |  | ND2 | MT241408 |
| H70 | 5238WS | CR | MT241498 |
|  |  | ND2 | MT241407 |

**Table S4** Migrate-n model testing for CR among the sampling area under 10000 and 50000 steps. The model with highest support is highlighted in grey.

| **Area 1** | **Area 2** | **Model** | **10000 steps** | | | **50000 steps** | | | |
| --- | --- | --- | --- | --- | --- | --- | --- | --- | --- |
|  |  |  | **Bezier log marginal-likelihood** | **Model choice** | **Probability** | **Bezier log marginal-likelihood** | **Model choice** | **Probability** |  |
| West Peninsular | East Peninsular | 1 ↔ 2 | -1709.9787 | 2 | 0.175 | -1710.8521 | 3 | 0.082 |  |
|  |  | 1 → 2 | -1710.4303 | 3 | 0.111 | -1709.5954 | 2 | 0.287 |  |
|  |  | **1 ← 2** | **-1708.5717** | **1** | **0.714** | **-1708.8065** | **1** | **0.631** |  |
|  |  | 1 ≠ 2 | -1718.2982 | 4 | 0.000 | -1718.3863 | 4 | 0.000 |  |
| East Peninsular | Sarawak | 1 ↔ 2 | -1786.7451 | 3 | 0.016 | -1787.3 | 3 | 0.013 |  |
|  |  | **1 → 2** | **-1782.8882** | **1** | **0.755** | **-1783.1604** | **1** | **0.798** |  |
|  |  | 1 ← 2 | -1784.0819 | 2 | 0.229 | -1784.6019 | 2 | 0.189 |  |
|  |  | 1 ≠ 2 | -1797.5571 | 4 | 0.000 | -1798.49 | 4 | 0.000 |  |
| Sarawak | Western Sabah | 1 ↔ 2 | -1785.6613 | 2 | 0.207 | -1785.153 | 2 | 0.124 |  |
|  |  | **1 → 2** | **-1784.5042** | **1** | **0.658** | **-1783.3423** | **1** | **0.759** |  |
|  |  | 1 ← 2 | -1786.1261 | 3 | 0.130 | -1785.2441 | 3 | 0.113 |  |
|  |  | 1 ≠ 2 | -1789.4857 | 4 | 0.005 | -1788.8349 | 4 | 0.003 |  |
| Sarawak | Eastern Sabah | 1 ↔ 2 | -1872.345 | 2 | 0.155 | -1873.0733 | 3 | 0.055 |  |
|  |  | **1 → 2** | **-1870.8107** | **1** | **0.719** | **-1870.3855** | **1** | **0.806** |  |
|  |  | 1 ← 2 | -1872.5558 | 3 | 0.126 | -1872.1425 | 2 | 0.139 |  |
|  |  | 1 ≠ 2 | -1879.9294 | 4 | 0.000 | -1880.0059 | 4 | 0.000 |  |
| Western Sabah | Eastern Sabah | **1 ↔ 2** | **-1663.0592** | **1** | **0.453** | **-1663.093** | **1** | **0.686** |  |
|  |  | 1 → 2 | -1663.2217 | 2 | 0.385 | -1664.8741 | 3 | 0.116 |  |
|  |  | 1 ← 2 | -1664.3237 | 3 | 0.128 | -1664.5089 | 2 | 0.167 |  |
|  |  | 1 ≠ 2 | -1665.6524 | 4 | 0.034 | -1666.1815 | 4 | 0.031 |  |

**Table S5** Migrate-n model testing for ND2 among the sampling area under 10000 and 50000 steps. The model with highest support is highlighted in grey.

| **Area 1** | **Area 2** | **Model** | **10000 steps** | | | **50000 steps** | | | |
| --- | --- | --- | --- | --- | --- | --- | --- | --- | --- |
|  |  |  | **Bezier log marginal-likelihood** | **Model choice** | **Probability** | **Bezier log marginal-likelihood** | **Model choice** | **Probability** |  |
| West Peninsular | East Peninsular | 1 ↔ 2 | -1497.4689 | 3 | 0.009 | -1496.1999 | 3 | 0.048 |  |
|  |  | 1 → 2 | -1498.3637 | 4 | 0.003 | -1499.4082 | 4 | 0.002 |  |
|  |  | **1 ← 2** | **-1492.9423** | **1** | **0.787** | **-1493.3144** | **1** | **0.868** |  |
|  |  | 1 ≠ 2 | -1494.3053 | 2 | 0.201 | -1495.675 | 2 | 0.082 |  |
| East Peninsular | Sarawak | 1 ↔ 2 | -1504.631 | 3 | 0.044 | -1504.3759 | 3 | 0.070 |  |
|  |  | **1 → 2** | **-1501.6496** | **1** | **0.874** | **-1501.9761** | **1** | **0.775** |  |
|  |  | 1 ← 2 | -1504.0231 | 2 | 0.081 | -1503.5911 | 2 | 0.154 |  |
|  |  | 1 ≠ 2 | -1509.1066 | 4 | 0.001 | -1508.5453 | 4 | 0.001 |  |
| Sarawak | Western Sabah | 1 ↔ 2 | -1540.1492 | 4 | 0.000 | -1540.5123 | 4 | 0.000 |  |
|  |  | 1 → 2 | -1540.1011 | 3 | 0.000 | -1539.7858 | 3 | 0.000 |  |
|  |  | 1 ← 2 | -1539.1919 | 2 | 0.001 | -1538.837 | 2 | 0.001 |  |
|  |  | **1 ≠ 2** | **-1532.4302** | **1** | **0.998** | **-1532.1772** | **1** | **0.998** |  |
| Sarawak | Eastern Sabah | 1 ↔ 2 | -1492.6311 | 2 | 0.052 | -1492.5943 | 3 | 0.072 |  |
|  |  | **1 → 2** | **-1489.7948** | **1** | **0.884** | **-1490.1822** | **1** | **0.805** |  |
|  |  | 1 ← 2 | -1492.942 | 3 | 0.038 | -1492.2727 | 2 | 0.100 |  |
|  |  | 1 ≠ 2 | -1493.3195 | 4 | 0.026 | -1493.7313 | 4 | 0.023 |  |
| Western Sabah | Eastern Sabah | 1 ↔ 2 | -1493.3713 | 4 | 0.084 | -1492.3336 | 3 | 0.123 |  |
|  |  | **1 → 2** | **-1491.2752** | **1** | **0.686** | **-1490.6243** | **1** | **0.679** |  |
|  |  | 1 ← 2 | -1493.1264 | 3 | 0.108 | -1492.9187 | 4 | 0.068 |  |
|  |  | 1 ≠ 2 | -1492.9985 | 2 | 0.122 | -1492.2771 | 2 | 0.130 |  |


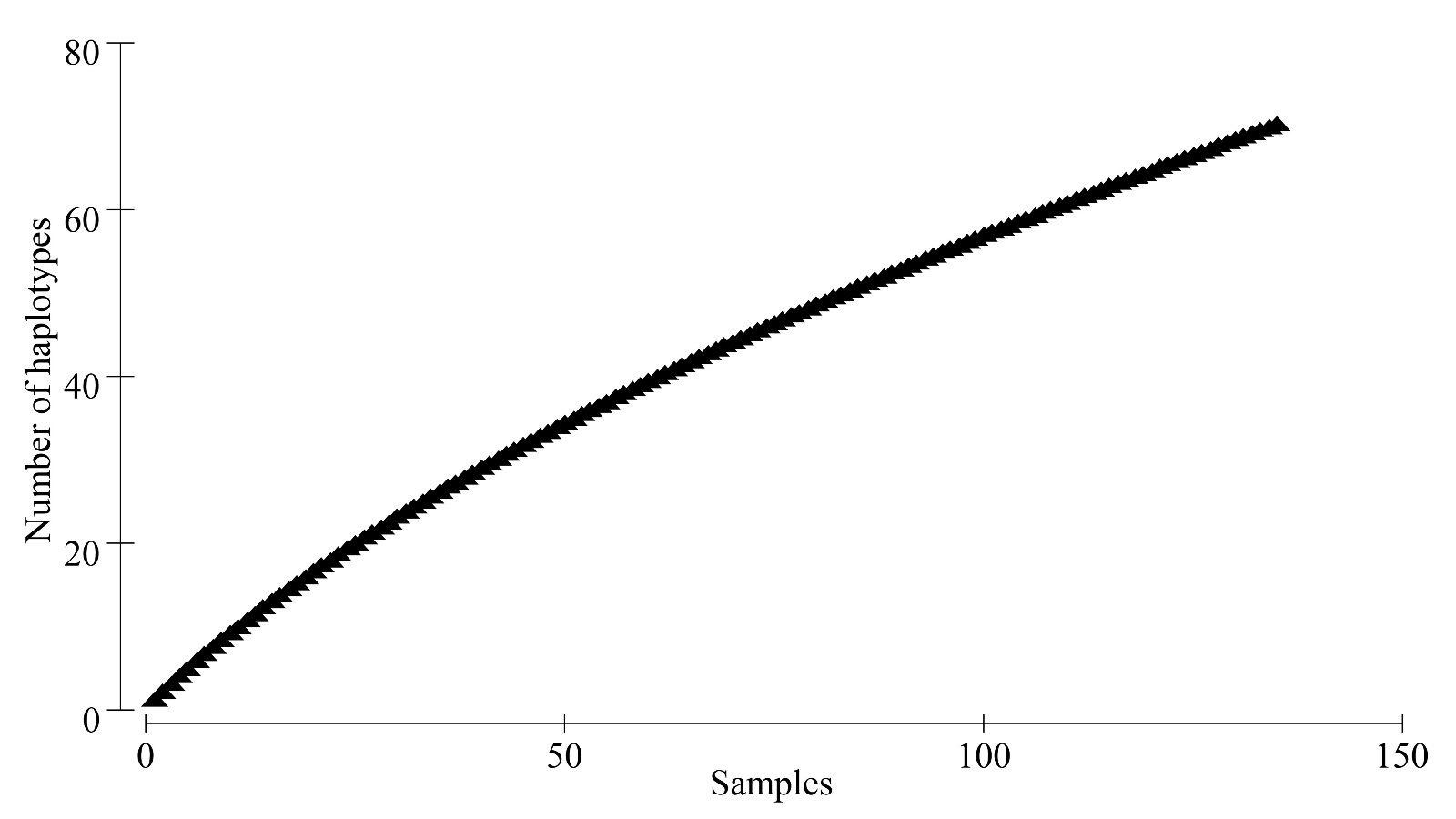


**Figure S1** Cumulative haplotype curves representing number of haplotype and samples size
